# Dissecting the contributions of organic nitrogen aerosols to global atmospheric nitrogen deposition and implications for ecosystems

**DOI:** 10.1093/nsr/nwad244

**Published:** 2023-09-18

**Authors:** Yumin Li, Tzung-May Fu, Jian Zhen Yu, Xu Yu, Qi Chen, Ruqian Miao, Yang Zhou, Aoxing Zhang, Jianhuai Ye, Xin Yang, Shu Tao, Hongbin Liu, Weiqi Yao

**Affiliations:** Shenzhen Key Laboratory of Precision Measurement and Early Warning Technology for Urban Environmental Health Risks, School of Environmental Science and Engineering, Southern University of Science and Technology, Shenzhen518055, China; Guangdong Provincial Observation and Research Station for Coastal Atmosphere and Climate of the Greater Bay Area, Southern University of Science and Technology, Shenzhen518055, China; Division of Environment and Sustainability, Hong Kong University of Science and Technology, Hong Kong999077, China; Shenzhen Key Laboratory of Precision Measurement and Early Warning Technology for Urban Environmental Health Risks, School of Environmental Science and Engineering, Southern University of Science and Technology, Shenzhen518055, China; Guangdong Provincial Observation and Research Station for Coastal Atmosphere and Climate of the Greater Bay Area, Southern University of Science and Technology, Shenzhen518055, China; National Center for Applied Mathematics Shenzhen, Shenzhen518055, China; Division of Environment and Sustainability, Hong Kong University of Science and Technology, Hong Kong999077, China; Department of Chemistry, Hong Kong University of Science and Technology, Hong Kong999077, China; Division of Environment and Sustainability, Hong Kong University of Science and Technology, Hong Kong999077, China; State Key Joint Laboratory of Environmental Simulation and Pollution Control, International Joint Laboratory for Regional Pollution Control, College of Environmental Sciences and Engineering, Peking University, Beijing100871, China; State Key Joint Laboratory of Environmental Simulation and Pollution Control, International Joint Laboratory for Regional Pollution Control, College of Environmental Sciences and Engineering, Peking University, Beijing100871, China; Frontier Science Center for Deep Ocean Multispheres and Earth System and Physical Oceanography Laboratory, Ocean University of China, Qingdao266100, China; College of Oceanic and Atmospheric Sciences, Ocean University of China, Qingdao266100, China; Shenzhen Key Laboratory of Precision Measurement and Early Warning Technology for Urban Environmental Health Risks, School of Environmental Science and Engineering, Southern University of Science and Technology, Shenzhen518055, China; Guangdong Provincial Observation and Research Station for Coastal Atmosphere and Climate of the Greater Bay Area, Southern University of Science and Technology, Shenzhen518055, China; Shenzhen Key Laboratory of Precision Measurement and Early Warning Technology for Urban Environmental Health Risks, School of Environmental Science and Engineering, Southern University of Science and Technology, Shenzhen518055, China; Guangdong Provincial Observation and Research Station for Coastal Atmosphere and Climate of the Greater Bay Area, Southern University of Science and Technology, Shenzhen518055, China; Shenzhen Key Laboratory of Precision Measurement and Early Warning Technology for Urban Environmental Health Risks, School of Environmental Science and Engineering, Southern University of Science and Technology, Shenzhen518055, China; Guangdong Provincial Observation and Research Station for Coastal Atmosphere and Climate of the Greater Bay Area, Southern University of Science and Technology, Shenzhen518055, China; Shenzhen Key Laboratory of Precision Measurement and Early Warning Technology for Urban Environmental Health Risks, School of Environmental Science and Engineering, Southern University of Science and Technology, Shenzhen518055, China; Guangdong Provincial Observation and Research Station for Coastal Atmosphere and Climate of the Greater Bay Area, Southern University of Science and Technology, Shenzhen518055, China; Department of Ocean Science, Hong Kong University of Science and Technology, Hong Kong999077, China; Department of Ocean Science and Engineering, Southern University of Science and Technology, Shenzhen518055, China

**Keywords:** organic nitrogen aerosol, secondary organic nitrogen, biomass burning

## Abstract

Atmospheric deposition of particulate organic nitrogen (ON_p_) is a significant process in the global nitrogen cycle and may be pivotally important for N-limited ecosystems. However, past models largely overlooked the spatial and chemical inhomogeneity of atmospheric ON_p_ and were thus deficient in assessing global ON_p_ impacts. We constructed a comprehensive global model of atmospheric gaseous and particulate organic nitrogen (ON), including the latest knowledge on emissions and secondary formations. Using this model, we simulated global atmospheric ON_p_ abundances consistent with observations. Our estimated global atmospheric ON deposition was 26 Tg N yr^−1^, predominantly in the form of ON_p_ (23 Tg N yr^−1^) and mostly from wildfires (37%), oceans (22%) and aqueous productions (17%). Globally, ON_p_ contributed as much as 40% to 80% of the total N deposition downwind of biomass-burning regions. Atmospheric ON_p_ deposition thus constituted the dominant external N supply to the N-limited boreal forests, tundras and the Arctic Ocean, and its importance may be amplified in a future warming climate.

## Teaser

Particulate organic nitrogen made up 19% of global atmospheric total N deposition and dominated the external N supply to N-limited ecosystems.

## INTRODUCTION

Organic nitrogen (ON) refers to the nitrogen (N) atoms covalently bound to organic molecules. ON in the atmosphere includes a wide variety of reduced and oxidized species [1,2] and has profound impacts on the biogeochemical cycle of nitrogen and on climate [3–8]. Atmospheric deposition of total N (TN), including both inorganic nitrogen (IN) and ON components, is estimated to be 77 to 135 Tg N yr^−1^ [9–11] and constitutes an important external source of nutrients to terrestrial and marine ecosystems. Observations have shown that, on average, 25% of the atmospheric TN deposition is organic, primarily in the form of particulate ON (ON_p_) (Supplementary Material: data sets S1 and S3). There are large regional and seasonal variations in the organic fractions of atmospheric TN deposition, yet the reasons for these variations are not well understood [2,12]. Moreover, the bioavailability of ON_p_ species to different terrestrial and marine primary producers ranges widely between 2% and 80% [6,13–17], while chronic exposure to some atmospheric ON_p_ species (e.g. quinoline) is toxic to terrestrial plants and marine plankton [1]. Atmospheric ON_p_ is also thought to be the dominant colored component of atmospheric brown carbon aerosol [18,19], affecting the radiative balance of Earth's climate system [8,20]. However, the global environmental impacts of atmospheric ON_p_ remain underdiagnosed, because their global sources, abundances, compositions and depositions are not well quantified.

**Figure 1.**
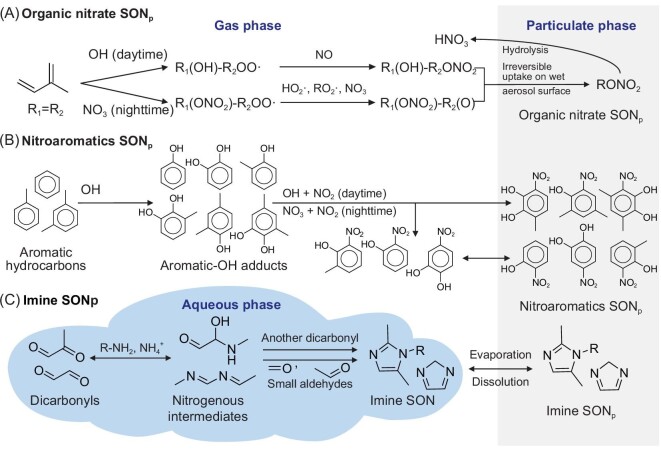
Schematic diagram of the three particulate secondary ON (SON_p_) formation pathways included in this study. (A) SON_p_ as part of the organic nitrates (RONO_2_) produced by the oxidation of volatile organic compounds by OH or NO_3_ radicals in the presence of NO*_x_*. (B) SON_p_ as part of the nitroaromatics (NACs) produced by the oxidation of aromatic compounds by OH and NO_3_ radicals in the presence of NO*_x_*. (C) SON_p_ as part of the imine-like compounds produced by the aqueous reactions of dicarbonyls with ammonium or amines. One-way arrows indicate irreversible reactions from precursors to products; double arrows indicate reversible reactions.

Atmospheric ON_p_ may be directly emitted from anthropogenic [21] and biomass-burning [22] activities and as constituent substances present in sea spray aerosol [23], dust [24] and primary biological atmospheric particulates [23], collectively referred to as primary ON_p_ (PON_p_). In addition, secondary ON_p_ (SON_p_) may be produced in the atmosphere from multiple pathways, including most importantly (Fig. 1): (i) via the gas-phase oxidation of aliphatic volatile organic compounds (VOCs) by OH or NO_3_ radicals in the presence of nitrogen oxides (NO*_x_* ≡ NO + NO_2_) to form semi-volatile organic nitrates, which are then irreversibly up-taken at the surface of wet aerosols [25,26]; (ii) via the gas-phase oxidation of aromatic VOCs by OH or NO_3_ radicals in the presence of NO*_x_* to form semi-volatile nitroaromatics, which partition into the particulate phase [27–29]; and (iii) via the aqueous reactions of dicarbonyls with ammonium or amines in cloud droplets and wet aerosols to form heterocyclic compounds with imine or amine functional groups (e.g. imidazoles, imidazole-2-carboxaldehyde and pyrroles) [30–34]. Previous modeling studies estimated global PON_p_ abundances by scaling primary organic carbon aerosol with observed N : C molar ratios, but those studies did not distinguish the N : C ratios from different biomass-burning and anthropogenic sources [10,11,35,36]. Previous studies also estimated SON_p_ by scaling secondary organic aerosol (SOA) with N : C ratios, by scaling ammonium abundance, or by simulating the simple formation of organic nitrates from the oxidation of biogenic VOCs at prescribed yields [10,11,35,36]. Their simulated atmospheric ON_p_ deposition fluxes underpredicted the observations by an order of magnitude at sites with high ON deposition fluxes, unless *ad hoc* scaling was applied [10,11,35,36]. Furthermore, previous studies have not evaluated their simulated atmospheric ON_p_ concentrations against measurements.

We present here a comprehensive global simulation of atmospheric ON for the year 2016, built on the GEOS-Chem 3-D chemical transport model (v12.9.3, http://geos-chem.org) at 5^o^ longitude × 4^o^ latitude resolution [37]. We incorporated the current-best knowledge of the primary sources of gaseous ON (ON_g_) and ON_p_ species and their N : C mass ratios, and the explicit formation pathways of SON_p_ (Fig. 1), as well as the chemical aging processes of ON_g_ and ON_p_ in the atmosphere (Materials and Methods; Supplementary Material: Text S1). We evaluated our simulated atmospheric ON_p_ abundances and deposition fluxes against global measurements and analyzed the simulated spatiotemporal and source variabilities of atmospheric ON_p_ deposition, with the goal of better quantifying the resulting external N supply to global ecosystems.

## RESULTS

### Simulated global atmospheric ON_p_ abundance, spatiotemporal variability and source attribution

**Figure 2.**
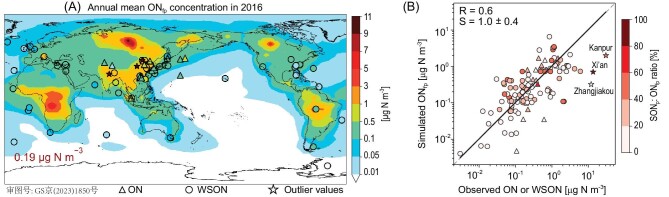
Observed and simulated global annual mean surface ON_fp_ concentrations. Observations shown as symbols. Triangles: ON measurements; circles: water-soluble ON (WSON) measurements; stars: outliers affected by strong local, intermittent sources. (A) Observed (symbols) and simulated (filled contours) annual mean surface ON_fp_ concentrations. The simulated global annual mean surface ON_fp_ concentration is shown inset. (B) Scatterplot of simulated versus observed annual mean surface ON_fp_ concentrations, color-coded by the SON_p_ : ON_fp_ ratios. The black line indicates the reduced major axis regression line, excluding the three outliers. The gray dashed line indicates the 1 : 1 line. The slope (S) and correlation coefficient (R) are shown inset.

Figures 2, S1 and S2 compare our simulated global annual mean surface concentrations of ON_p_ and fine ON_p_ (particulate ON with aerodynamic diameters <2.5 μm, ON_fp_) against observations at global surface sites between the years 1999 and 2020 (Supplementary Material: data set S1). We combined measurements of ON_p_ and the water-soluble part of ON_p_ (WSON_p_) reported in the literature, because measurements showed most ON_p_ to be soluble, except for the ON_p_ sampled at some marine and dust-affected sites. We also focus below on the evaluation of simulated ON_fp_, because most measurements only sampled fine particles; in the few studies where both fine and coarse particles were measured, the observed ON_p_ was predominantly in the fine particles. Observed ON_fp_ concentrations at global sites ranged between 0.01 and 30 μg N m^−3^ (global average 0.47 μg N m^−3^, excluding three extremely high ON_fp_ observations affected by local sources), with higher concentrations at sites affected by biomass-burning and anthropogenic sources and generally lower concentrations at remote marine sites. Our simulated ON_fp_ concentrations matched the observed magnitudes and spatial distributions of surface ON_fp_ with no apparent systematic biases (Fig. 2B, slope = 1.0 ± 0.4, correlation coefficient *R* = 0.6). We also evaluated our simulated ON_fp_ against seasonal measurements in Southern China and found that the model reproduced the observed ON_fp_ concentrations and the enhancements during the cold season (Fig. S3, data set S1). Thus, our simulation represented the first successful model depiction of observed global surface ON_fp_ abundance. The model underestimated three extremely high ON_fp_ observations, which were sampled during local incineration events of agricultural residues and wastes (Kanpur, India) [38] or during local dust storms (Xi’an and Zhangjiakou, China) [24,39]. These local, intermittent emitting events were either under-represented in the satellite-based burning activity data or missed by our global simulation for a different year.

The observed surface ON_p_ concentrations were similar to those of ON_fp_ (Fig. S2), because ON_p_ were mostly in the fine mode. Our simulated global annual mean surface ON_p_ concentration was 0.23 μg N m^−3^, also mostly in the form of ON_fp_ (0.19 μg N m^−3^, 83% of simulated ON_p_). Surface ON_fp_ were mostly from biomass-burning emissions (0.12 μg N m^−3^, 63%), secondary production (0.04 μg N m^−3^, 21%) and anthropogenic emissions (0.02 μg N m^−3^, 11%) (Figs S1, S4 and S5). The highest simulated annual mean ON_fp_ concentrations were over the Siberian and tropical forests (1 to 11 μg N m^−3^), reflecting the emissions of ON_fp_ and its precursors from wildfires. Simulated ON_fp_ concentrations exceeded 0.5 μg N m^−3^ over East Asia, South Asia and Southeast Asia, due to the pronounced anthropogenic emissions in these regions. Dust (0.003 μg N m^−3^) and marine (0.009 μg N m^−3^) contributions to ON_fp_ were relatively small because these sources emitted ON_p_ mainly as coarse particles.

SON_p_ contributed >20% of the simulated ON_fp_ concentrations at approximately one-third of the surface sites, especially at locations with high ON_fp_ concentrations (Figs 2B, S1 and S4). The simulated global SON_p_ predominantly consisted of imine SON_p_, produced via the aqueous reaction of dicarbonyls with ammonium (Figs S1 and S4). Simulated annual mean surface imine SON_p_ concentrations exceeded 0.1 μg N m^−3^ in East and South Asia, Southeast USA, the boreal forests, and the rainforests of Africa and South America, reflecting the emissions of dicarbonyls and their precursors from anthropogenic, biomass-burning and biogenic activities [40,41]. In our model, organic nitrate SON_p_ and nitroaromatics SON_p_ each contributed <0.001 μg N m^−3^ (0.5%) of the global mean surface ON_fp_, respectively. The spatial distributions of SON_p_ from all three secondary formation pathways were similar due to their common precursor sources (Fig. S4).

We further evaluated our simulated SON_p_ compositions against the limited measurements of particulate nitroaromatics and organic nitrates currently available (Supplementary Material: data set S2). Our simulated nitroaromatic SON_p_ concentrations (0.005 to 78 ng N m^−3^) were consistent with the observed abundance and spatial distribution of particulate nitroaromatics (0.09 to 250 ng N m^−3^, *R* = 0.6, Figs S4I and S6). Observations showed that molecules containing organic nitrate functional groups comprised 4% to 28% and 2% to 25% of the ambient surface organic aerosol mass in China [42–44] and in the USA [45,46], respectively (data set S2). Assuming a typical molecular weight of 250 g mole^−1^ (corresponding to organic nitrate molecules with N : C mass ratios of 0.1 to 0.5) [46], our simulated particulate organic nitrates comprised 3% to 27% and 5% to 24% of the simulated surface organic aerosol mass in China and in the USA, respectively, consistent with the observations. Our simulated organic nitrate SON_p_ concentrations (0.005 to 300 ng N m^−3^) were consistent within an order of magnitude against most particulate organic nitrate observations over North America and Asian sites, but the simulated concentrations were systematically lower than the observations in Europe (Figs S4H and S7). However, those mass-spectrometry-based particulate organic nitrate measurements in Europe might have been biased towards being high, because the researchers attributed a larger fraction of the total detected nitrate fragments to organic nitrates by assuming a large NO_2_^+^ to NO^+^ fragment ratio for organic nitrates (0.1) [47,48]. On the other hand, it is also possible that our simulated particulate organic nitrate concentrations are biased towards being low. Observations showed that particulate organic nitrates in Europe were mostly formed from night-time biogenic VOC oxidation by NO_3_ [47]. Chamber experiments and ambient measurements showed that the molar yields of particulate organic nitrates from isoprene-NO_3_ oxidation were between 4% and 24% [49], while the molar yields of particulate organic nitrates from monoterpene-NO_3_ oxidation were between 15% and 57% [50,51]. In our simulation, the molar yields of gaseous organic nitrates from VOC-NO_3_ reactions and the uptake coefficient of gaseous organic nitrates by aqueous particles were fitted to the ambient observations in Southeast USA [26], resulting in overall global particulate organic nitrate yields of 22% from isoprene-NO_3_ reactions and 9% from monoterpenes-NO_3_ reactions. Thus, our simulated production of particulate organic nitrates from monoterpenes may be low and lead to organic nitrate underestimation over Europe, since that pathway was a larger contributor to organic nitrates in Europe than it was in China and the USA. Furthermore, we assumed that all particulate organic nitrates in the aqueous phase underwent hydrolysis to form nitric acid at a timescale of an hour, based on a fitting to ambient measurements in Southeast USA [26]. However, chamber experiments found that the lifetimes of organic nitrates against hydrolysis may vary between minutes and weeks, depending on the molecular structure of the organic nitrate species and the pH value of the solution [52–55]. In particular, some organic nitrates from monoterpenes with a ring skeleton containing three delocalized π orbitals hydrolyze slowly at lifetimes exceeding a week [54,55], while some non-tertiary nitrates from isoprene do not undergo hydrolysis at all [52,53]. In addition, recent chamber studies have demonstrated that gas-phase organic nitrates produced from monoterpene oxidation may photolyze at rates 2 to 10 times slower than the photolysis rates we assumed for organic nitrates from monoterpene (lifetimes against photolysis 1 to 2 hours) [56,57]; this potential bias may also contribute to our underestimation of particulate organic nitrates.

We evaluated the robustness of our global ON_p_ abundance estimates by conducting sensitivity experiments, in which the N : C ratios for PON_p_ emissions and SON_p_ formation rates were varied within their respective literature-reported ranges (Supplementary Material: Text S2). From these sensitivity experiments, the range of global annual mean surface ON_fp_ concentrations was between 0.06 and 0.32 μg N m^−3^ (ON_p_ concentrations between 0.08 and 0.36 μg N m^−3^, Figs S8 and S9). Coarse ON_p_ (ON particles >2.5 μm in diameter, ON_cp_) accounted for <25% of total ON_p_ concentration everywhere except at marine sites, and our varying the N : C emission ratios of primary ON_cp_ had very little impact on the simulated global surface ON_p_ concentrations. This was consistent with previous observations of atmospheric ON_p_ being predominantly in the fine mode (Supplementary Material: data set S1).

On a global scale, the most variable sources for our simulated ON_p_ were biomass-burning emissions and imine SON_p_ production. We found that a PON_p_-enhanced scenario (with high-end biomass-burning N : C emission ratio and low-end imine SON_p_ formation rates) and an SON_p_-enhanced scenario (with low-end biomass-burning N : C emission ratio and high-end imine SON_p_ formation rates) would both produce results similar to our standard simulation and consistent with observed surface ON_p_ concentrations (Figs S10 and S11). The simulated mean global surface ON_fp_ concentrations were 0.22 μg N m^−3^ for the PON_p_-enhanced scenario and 0.15 μg N m^−3^ for the SON_p_-enhanced scenario, respectively, with biomass burning contributing 64% and 7% of the mean global surface ON_fp_ abundance, and imine SON contributing 9% and 74% of the mean global surface ON_fp_ abundance, respectively. However, published observations were deficient in distinguishing these two scenarios, because there were relatively few measurements in areas strongly affected by biomass burning and no explicit measurements of imine SON_p_ (Figs 2 and S1). As a result, our standard simulation may underestimate the contributions of biomass burning PON_p_ or SON_p_ to global ON_p_ abundance, potentially up to a factor of 9.

### Evaluation of simulated atmospheric ON deposition flux

**Figure 3.**
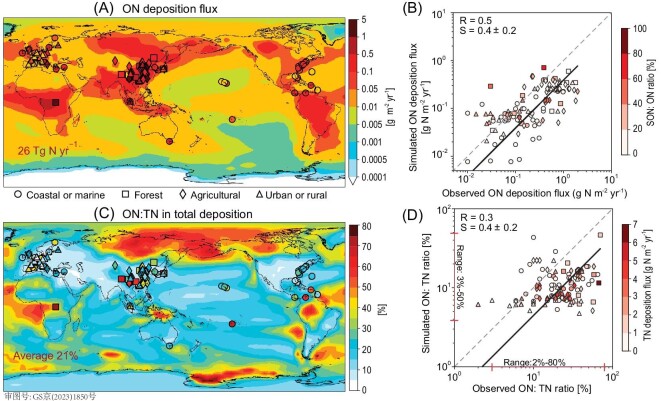
Observed and simulated atmospheric ON deposition fluxes and the ON : TN ratios in atmospheric deposition fluxes. (A) Observed (symbols) and simulated (filled contours) ON deposition fluxes. (B) Scatterplot of (A). (C) Observed and simulated ON : TN ratios in atmospheric deposition fluxes. (D) Scatterplot of (C). The black lines indicate the reduced major axis regression lines; the gray dashed lines show the 1 : 1 lines. The symbol colors in (B) indicate the simulated SON mass fraction in the total ON deposition flux at each site. The symbol colors in (D) indicate the simulated TN deposition fluxes.

Figure 3 evaluates the simulated atmospheric deposition fluxes of ON (including ON_g_ and ON_p_) and the ON: TN ratios in those deposited fluxes against global surface observations (Supplementary Material: data set S3). Observations of ON deposition fluxes were subject to significant uncertainty. One-third of the published ON deposition measurements only analyzed dissolved ON (DON) contents and only in rainwater samples, thus they might under-represent the atmospheric deposition of ON. On the other hand, ON contents were almost always inferred by the measured differences between TN and IN. This technique tended to overestimate ON deposition and its ON : TN ratios, particularly where ON deposition fluxes were low because negative ON measurement was either rounded up to zero or excluded [58,59]. We compared our model results to all published measurements but noted these technical issues as potential causes for discrepancies between the model and the observations.

**Figure 4.**
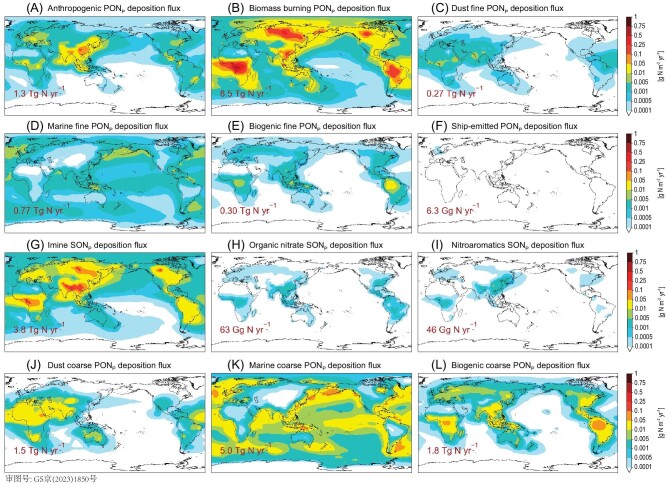
Simulated annual mean atmospheric deposition fluxes of ON_p_ from different sources. (A) Anthropogenic PON_p_. (B) Biomass burning PON_p_. (C) Fine dust PON_p_. (D) Fine marine PON_p_. (E) Fine biogenic PON_p_. (F) Ship-emitted PON_p_. (G) Imine SON_p_. (H) Organic nitrate SON_p_. (I) Nitroaromatic SON_p_. (J) Coarse dust PON_p_. (K) Coarse marine PON_p_. (L) Coarse biogenic PON_p_.

Our simulated global atmospheric ON deposition flux was 26 Tg N yr^−1^ (including 2.5 Tg N yr^−1^ of ON_g_ and 23 Tg N yr^−1^ of ON_p_), and the spatial distribution of simulated fluxes was consistent with the observed gradients of ON deposition fluxes from marine (0.01 to 0.99 g N m^−2^ yr^−1^) to inland (0.07 to 3.8 g N m^−2^ yr^−1^) sites (Fig. 3). Over land, the model reproduced the observed high ON deposition fluxes over South and East Asia, Western Europe, the tropical forests of Africa and South America, and the boreal forests of North America and Siberia, reflecting the deposition of atmospheric ON from biomass-burning and anthropogenic sources (Figs 3 and 4). Over the ocean, the observed and simulated ON deposition fluxes both showed enhancements downwind of areas with pronounced biomass-burning, anthropogenic and dust emissions, as well as over locations with enhanced marine ON emissions. Overall, our simulated ON deposition fluxes were lower than the observed ON deposition fluxes by a factor of three (Fig. 3B), an improvement over previous model studies that underestimated the observed ON deposition fluxes by more than one order of magnitude, especially at sites with high ON deposition fluxes [10,11,35,36]. This improved representation of atmospheric ON deposition relative to previous studies was driven by a combination of model improvements: our use of updated, source-specific N : C ratios for primary ON_fp_ from biomass burning and anthropogenic combustion, the use of updated N : C ratios for ON_cp_ from marine and dust emissions, as well as the explicit inclusion of imine SON_p_ formations. Varying the N : C emission ratios for ON_cp_ within the literature-reported ranges led to simulated global ON_cp_ deposition fluxes between 4.5 and 8.9 Tg N yr^−1^ (Supplementary Material: Text S2); our selected high-end N : C emission ratios for ON_cp_ conformed with the observed deposition fluxes. Our simulation showed that SON_p_ contributed >20% of the atmospheric ON deposition fluxes in one-third of the observed sites, particularly at forested, urban and rural locations.

Figure 3C and D compare the simulated and observed ON : TN ratios in the atmospheric deposition fluxes. The observed ON : TN ratios averaged 25% globally but showed wide-ranging regional variability between 2% and 70% (Supplementary Material: data set S3). Our simulated ON : TN deposition ratios at the observation sites ranged between 3% and 50% with a global average of 21%. Observed ON : TN ratios in deposition fluxes at a single site varied by a factor of two to five, partially reflecting the measurement uncertainties described above and partially reflecting the interannual variation of observations. On a site-by-site basis, the discrepancies between our simulated ON : TN ratios and the observations were mostly within a factor of five (Fig. 3D). Therefore, our simulated ON : TN ratios agreed with the observations within the uncertainties of observations. These comparisons represented the first site-by-site evaluation of global simulated ON : TN deposition ratios and indicated that our model was capable of simulating the atmospheric N deposition fluxes and the ON contributions to global ecosystems.

To test the robustness of our simulations, we analyzed the results from the sensitivity experiments where N : C ratios for PON_p_ and production rates of SON_p_ were varied within their literature-reported ranges (Supplementary Material: Text S2; Figs S13–S16). In these sensitivity experiments, the simulated global ON deposition flux ranged between 10 and 40 Tg N yr^−1^, with ON contributing 9% to 29% of the global TN deposition. SON_p_ contributed 6% to 61% of the total ON deposition. We found that the experiment with an upper-limit N : C ratio for PON_p_ and the fastest production for SON_p_ still underestimated the observed ON deposition fluxes by a factor of two (Fig. S13), especially in the high-ON_p_ regions. These discrepancies indicated potential underestimation of the biomass-burning and anthropogenic emissions of PON_p_ or SON_p_ precursors.

We conducted further sensitivity tests to fit the simulated ON deposition fluxes against the observations by increasing the PON_p_ emissions from anthropogenic and biomass-burning sources, and by increasing the imine SON_p_ production (Fig. S18). We found that increasing the anthropogenic PON_p_ emissions by a factor of nine would result in good agreement between the simulated and observed ON deposition fluxes but would lead to an overestimation of surface ON_fp_ abundance by a factor of four. In contrast, increasing biomass-burning PON_p_ emissions or imine SON_p_ production by a factor of five, respectively, would both result in good agreement between the simulated and observed ON deposition fluxes, while the simulated surface ON_fp_ concentrations would only be larger than current observations by a factor of two. These findings again confirmed that ON_p_ from biomass burning, and secondary productions, may be biased towards being low in our standard simulation, and that further measurements representing these sources are needed to better constrain the global abundance and deposition fluxes of ON.

### Global budget of atmospheric ON and contribution to atmospheric TN deposition

Table 1 summarizes the global budget of atmospheric ON as simulated by our model. The total atmospheric burden of ON was 1.3 Tg N (range in sensitivity experiments was 1.1 Tg N to 1.5 Tg N), including 1.0 Tg N of ON_g_ and 0.3 Tg N of ON_p_. ON_g_ species were mostly chemically produced in the atmosphere as acyl peroxy nitrates (e.g. peroxyacetyl nitrate) and non-acyl peroxy nitrates (e.g. methyl peroxy nitrate), and all ON_g_ species had limited solubility [2]. As such, ON_g_ were mainly removed from the atmosphere by thermal decomposition, photolysis or OH oxidation [26,60], with deposition accounting for a mere 1% to 2% of its global sink [61]. Globally, ON_g_ only constituted 9% of the total atmospheric ON deposition. In contrast, ON_p_ constituted only 23% of the global atmospheric ON burden but dominated the global atmospheric ON deposition (91%). Of the 0.3 Tg N global atmospheric ON_p_ burden, 87% (0.26 Tg N) was in the fine mode (ON_fp_). ON_cp_ constituted only 13% (0.04 Tg N) of the global ON_p_ burden because of its rapid deposition. Globally, biomass-burning (8.5 Tg N yr^−1^) and anthropogenic (1.3 Tg N yr^−1^) emissions were the most important primary sources of ON_fp_, while marine emissions were the dominant primary source of ON_cp_. Net secondary production constituted an atmospheric ON_p_ source of 3.9 Tg N yr^−1^, 97% of which was associated with the aqueous reaction of dicarbonyls with ammonium to form imine SON_p_.

**Table 1.** Global budget of atmospheric ON and the ON : TN ratios in atmospheric deposition as simulated by the GEOS-Chem model.

Source types	Atmospheric burden [Tg N]	Emission [Tg N yr^−1^]	Net chemical production^a^ [Tg N yr^−1^]	Dry deposition^b^ [Tg N yr^−1^]	Wet deposition^b^ [Tg N yr^−1^]	Total deposition^b^ [Tg N yr^−1^]	Total deposition to the ocean^b^ [Tg N yr^−1^]
**Total ON (ON_p_ + ON_g_)**	1.3	20	5.8	6.0 (5.0)	20 (17)	26 (22)	11 (8.0)
**Particulate ON (ON_p_)**	0.31	19	3.9	4.0 (3.0)	19 (17)	23 (20)	10 (7.5)
***Fine mode* (ON_fp_)**							
Anthropogenic emissions	0.021	1.3	–	0.29 (0.23)	1.0 (1.0)	1.3 (1.2)	0.51 (0.47)
Biomass-burning emissions	0.16	8.5	–	1.7 (1.4)	6.7 (6.5)	8.5 (7.9)	2.4 (2.3)
Dust emissions	0.0079	0.27	–	0.059 (0.042)	0.21 (0.20)	0.27 (0.24)	0.11 (0.10)
Primary biological particle emissions	0.0070	0.30	–	0.053 (0.053)	0.25 (0.25)	0.30 (0.30)	0.074 (0.074)
Ship emissions	0.000 062	0.0063	–	0.0016 (0.0012)	0.0047 (0.0043)	0.0063 (0.0055)	0.0049 (0.0049)
Marine emissions	0.0032	0.77	–	0.22 (0.12)	0.55 (0.45)	0.77 (0.57)	0.65 (0.46)
Organic nitrate SON_p_	0.00 080	–	0.069	0.028 (0.028)	0.040 (0.040)	0.068 (0.068)	0.016 (0.016)
NAC SON_p_	0.00 082	–	0.046	0.011 (0.011)	0.035 (0.035)	0.046 (0.046)	0.015 (0.015)
Imine SON_p_	0.065	–	3.8	0.56 (0.56)	3.2 (3.2)	3.8 (3.8)	0.98 (0.98)
***Coarse mode* (ON_cp_)**							
Dust emissions	0.023	1.5	–	0.22 (0.14)	1.3 (1.0)	1.5 (1.1)	0.44 (0.40)
Primary biological particle emissions	0.0056	1.8	–	0.13 (0.13)	1.7 (1.7)	1.8 (1.8)	0.33 (0.33)
Marine emissions	0.014	5.0	–	0.63 (0.31)	4.4 (2.3)	5.0 (2.6)	4.6 (2.4)
**Gaseous ON (ON_g_)**	1.0	0.75	1.7	2.0 (2.0)	0.45 (0.41)	2.5 (2.5)	0.49 (0.49)
**ON:TN in deposited fluxes**	–	–	–	11% (10%)	28% (25%)	21% (18%)	23% (18%)

a Net chemical production indicates the net effect of chemical production and the loss process. ^b^Soluble ON fluxes shown in parentheses.

**Figure 5.**
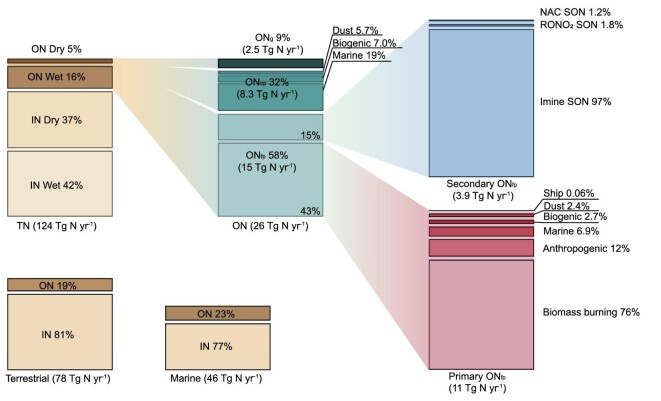
The global atmospheric TN deposition flux and the contributions of different N components as simulated by this study.

Figure 5 illustrates the contributions of different N components to the global atmospheric TN deposition. Our simulated global atmospheric TN deposition flux was 124 Tg N yr^−1^, including 78 Tg N yr^−1^ and 46 Tg N yr^−1^ to the global terrestrial and marine ecosystems, respectively. On a global scale, IN and ON each contributed 79% (98 Tg N yr^−1^) and 21% (26 Tg N yr^−1^) of the atmospheric TN deposition, respectively. Deposition of atmospheric ON was mostly through wet scavenging (20 Tg N yr^−1^) and less through dry deposition (6.0 Tg N yr^−1^), because the dominant depositing component, ON_p_, was highly water soluble. In contrast, the dry and wet deposition fluxes of IN were comparable in magnitudes, because the dry deposition of gaseous IN species was more efficient than that of gaseous ON species. Overall, the atmospheric deposition fluxes of ON_p_ (23 Tg N yr^−1^) constituted 19 Tg N yr^−1^ PON_p_ (including 8.5 Tg N yr^−1^ from biomass burning, 5.0 Tg N yr^−1^ from marine emissions and 1.3 Tg N yr^−1^ from anthropogenic emissions) and 3.9 Tg N yr^−1^ SON_p_, each contributing 83% and 17% of the global atmospheric ON_p_ deposition, respectively. Primary and secondary ON_p_ each constituted 16% and 3% of the global atmospheric TN deposition, respectively.

Previous model estimates for the atmospheric ON deposition fluxes, without applying *ad hoc* scaling, were between 10 and 32 Tg N yr^−1^, but their simulated atmospheric ON deposition fluxes were lower than observations by one order of magnitude, especially at high-ON locations [10,11,35,36]. In comparison to our simulated global ON budget, we found that the discrepancy between previous model studies and observations arose from two aspects. Firstly, previous model studies emitted larger amounts of ON_p_ from marine sources and primary biological aerosol particles (PBAPs), such that more than half of their global atmospheric ON_p_ deposition was due to the deposition of these natural ON_p_ species, but their simulated deposition fluxes were still lower than the observations at the high-ON locations affected by anthropogenic and biomass-burning activities. Secondly, previous studies estimated a larger deposition flux of ON_g_, because they assumed all ON_g_ species were soluble. As such, previous studies attributed 30% of global atmospheric ON deposition to ON_g_ [10]. In terms of the origins of ON_fp_, previous assessments estimated the combined deposition flux of anthropogenic and biomass burning PON_p_ to be 8 to 15 Tg N yr^−1^, with PON_p_ being largely from anthropogenic sources [10,11,35,36]. We showed that the global abundance and deposition of PON_p_ were predominantly from biomass burning, for which our estimated emissions may still be too low. Increasing the anthropogenic source of PON_p_ in our model to match the observed deposition fluxes led to severe overestimation of the observed surface ON_p_ concentrations (Fig. S18). In addition, previous studies estimated the atmospheric deposition flux of SON_p_ to be 2 to 18 Tg N yr^−1^, 30% to 100% of which consisted of oxidized SON_p_ [10,11,35,36]. Our simulation indicated that the global atmospheric SON_p_ was predominantly imine-like, reduced ON species, which was produced from the aqueous-phase reactions of dicarbonyls and ammonium.

### Impacts of spatially inhomogeneous atmospheric ON_p_ deposition on global ecosystems

Our simulation shows that the ON : TN ratios in atmospheric deposition fluxes have strong geographical variabilities that are closely related to the regional sources of ON, which would also affect the chemical composition of deposited ON. Global terrestrial and marine ecosystems are distinctly limited by N or other nutrients [4,62]. In addition, laboratory studies showed that the bioavailability of different ON species, i.e. the percentage of ON mass that can be assimilated by primary producers, ranged between 2% and 80% for bulk PON_p_ from different sources with large uncertainty [6,13–17], while the reduced ON_p_ species (e.g. imines) were almost entirely bioavailable (Supplementary Material: Text S3) [63]. Therefore, the ecological impacts of atmospheric ON deposition may be regionally disparate.

**Figure 6.**
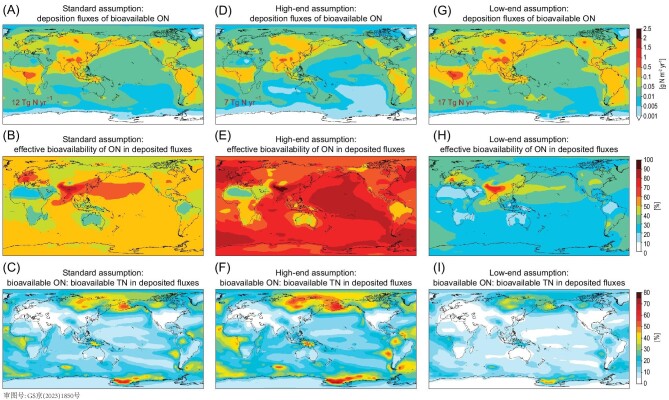
Spatial distribution of bioavailable ON deposition fluxes from the atmosphere. (A, D and G) Simulated atmospheric deposition fluxes of bioavailable ON. (B, E and H) The effective bioavailability of ON in the deposited fluxes. (C, F and I) The ratios of bioavailable ON versus bioavailable TN in the deposition fluxes. The left column shows the results from our standard assumption on ON bioavailability. The middle and right columns show the results assuming high-end and low-end values of ON bioavailability, respectively (Supplementary Material: Text S3). The global atmospheric bioavailable ON deposition fluxes are shown inset.

Our simulated spatial and chemical inhomogeneity of atmospheric ON deposition indeed led to great variability in the effective bioavailability of ON deposited to global ecosystems (Fig. 6; Supplementary Material: Text S3). Our calculations showed that atmospheric ON deposited over East Asia, Southeast Asia, Europe, the Pacific and North Atlantic were of higher bioavailability, because the ON deposited over these areas contained large fractions of imine SON_p_. In contrast, the atmospheric ON deposited over the arid areas of Africa, the Middle East, Australia and South America were predominantly from dust and of lower bioavailability (Fig. 4).

We highlight two types of regions: (i) regions near and downwind of biomass-burning emissions, and (ii) regions near and downwind of anthropogenic sources. Over ecosystems near and downwind of biomass-burning emissions, including the boreal forests and tundra, the tropical forests, the tropical Atlantic and the Arctic Ocean, the simulated atmospheric ON deposition fluxes exceeded 0.1 g N m^−2^ yr^−1^. The simulated deposited ON : TN ratios ranged from 40% to 80%, the highest values globally and consistent with the limited observations of atmospheric deposited ON : TN ratios at biomass-burning-affected forest sites (observed values between 19% and 70% with an average of 41%; Supplementary Material: data set S3). We calculated that the effective bioavailability of ON deposited over these areas was 50% (sensitivity calculations ranged from 34% to 87%), such that ON may potentially contribute 14% to 70% of the atmosphere-supplied bioavailable N into these ecosystems (Fig. 6; Supplementary Material: Text S3). The ecological impacts of atmospheric ON deposition may be pivotal over these areas, if N was the local limiting nutrient and other external N inputs were small. This finding was consistent with previous studies that showed that the biogeochemical cycling of N in primeval forests was mainly driven by the ON emissions and depositions associated with biomass burning [3,64], especially in boreal regions [6]. Our simulations further showed that—in these boreal forests, tundra and tropical forests affected by biomass burning—20% to 60% of the deposited ON_p_ were secondary and chemically reduced (Fig. 4). This finding suggests that atmospheric deposition of reduced ON_p_ may play an important role in the biogeochemistry of these ecosystems, especially in the N-limited boreal forest and tundra [62], but that the biogeochemical role of atmospheric ON has not been fully explored. Similarly, marine ecosystems in the Arctic Ocean and the tropical Atlantic are receptors of biomass burning ON_p_ transported long range from the boreal forest and Africa, respectively. The impacts of atmospheric ON deposition on productivity in these ecosystems are complex, as the biological assimilation of N in oceans is tightly coupled to other essential nutrients (such as iron and phosphorus) and temperature [4,65]. In the Arctic Ocean, where primary production is known to be N-limited in summer [66,67], atmospheric deposition of ON constituted a large external N source and may increase regional primary productivity. Microbial species capable of assimilating the deposited atmospheric ON potentially have a competitive advantage there [63,68].

Over regions strongly affected by anthropogenic sources, including East and South Asia, Europe, the Northwestern North Pacific and the North Indian Ocean, the simulated ON : TN ratios were typically below 20% due to the abundant anthropogenic IN in the regional atmosphere (Fig. 4). However, the simulated atmospheric ON deposition fluxes over these regions were still large (>0.1 g N m^−2^ yr^−1^), and 20% to 40% of the deposited ON fluxes were secondary and chemically reduced. Furthermore, the ON deposited over these regions were highly bioavailable (50% to 90% effective bioavailability, Fig. 6; Supplementary Material: Text S3). The marine ecosystems of the Northwestern Pacific and the North Indian Ocean are also known to be N-limited [4], such that the deposition of atmospheric ON, particularly the more bioavailable, reduced ON, may have large impacts there.

## DISCUSSION

Our simulated atmospheric ON_p_ abundance and ON deposition fluxes were consistent with most available observations, although there remained a factor-of-three discrepancy between our simulated global atmospheric ON deposition fluxes and observations. We showed that increases in the biomass-burning emissions of PON_p_ or the production of SON_p_ may help close that discrepancy, highlighting the need for targeted ambient measurements in biomass-burning-affected areas, and for information regarding the abundance and chemical composition of SON_p_, to better constrain these sources.

In a future warming climate, wildfires will likely intensify and become more frequent [69], increasing their emissions of both PON_p_ and precursors of SON_p_. Meanwhile, anthropogenic emissions of nitrogen oxides will continue to decrease in the future, reducing the abundance of oxidized IN in the atmosphere and its deposition [70]. A warming climate will also lead to more pronounced thermal stratification of the surface ocean, enhancing the importance of atmospheric N deposition as an external N source for surface marine ecosystems [71]. Atmospheric ON deposition may become an increasingly important external N source for global terrestrial and marine ecosystems, and its impact warrants further investigation.

## MATERIALS AND METHODS

Detailed descriptions of all methods and materials are presented in the Supplementary Material. Briefly, we developed a global atmospheric gaseous and particulate ON simulation for the year 2016 using the GEOS-Chem global 3-D chemical transport model (v12.9.3, http://geos-chem.org) [37] at a horizontal resolution of 5^o^ longitude × 4^o^ latitude and with 72 vertical layers. The simulation represented the primary emissions of atmospheric particulate and gaseous ON, the formation pathways of gaseous and particulate secondary ON in the atmosphere, and chemical aging of gaseous and particulate ON (Supplementary Material: Text S1). We conducted sensitivity simulations to evaluate the impacts of emission ratios and chemical parameters on the global budget of ON_p_ (Supplementary Material: Text S2). We calculated the bioavailability of atmospherically deposited ON fluxes to primary producers (Supplementary Material: Text S3). Published observations of atmospheric ON_p_ concentrations and atmospheric ON deposition fluxes are described and compiled in data sets S1, S2 and S3. Each data set includes a complete list of references to the observational studies.

## Supplementary Material

nwad244_Supplemental_Files

## Data Availability

All data are available in the main text or the Supplementary Materials. The GEOS-Chem model code used in this study is permanently archived at https://doi.org/10.57760/sciencedb.o00005.00024.
