# Correction to: Dissecting the contributions of organic nitrogen aerosols to global atmospheric nitrogen deposition and implications for ecosystems

**DOI:** 10.1093/nsr/nwaf538

**Published:** 2026-01-13

**Authors:** Yumin Li, Tzung-May Fu, Jian Zhen Yu, Xu Yu, Qi Chen, Ruqian Miao, Yang Zhou, Aoxing Zhang, Jianhuai Ye, Xin Yang, Shu Tao, Hongbin Liu, Weiqi Yao

**Affiliations:** Shenzhen Key Laboratory of Precision Measurement and Early Warning Technology for Urban Environmental Health Risks, School of Environmental Science and Engineering, Southern University of Science and Technology, Shenzhen 518055, China; Guangdong Provincial Observation and Research Station for Coastal Atmosphere and Climate of the Greater Bay Area, Southern University of Science and Technology, Shenzhen 518055, China; Division of Environment and Sustainability, Hong Kong University of Science and Technology, Hong Kong 999077, China; Shenzhen Key Laboratory of Precision Measurement and Early Warning Technology for Urban Environmental Health Risks, School of Environmental Science and Engineering, Southern University of Science and Technology, Shenzhen 518055, China; Guangdong Provincial Observation and Research Station for Coastal Atmosphere and Climate of the Greater Bay Area, Southern University of Science and Technology, Shenzhen 518055, China; National Center for Applied Mathematics Shenzhen, Shenzhen 518055, China; Division of Environment and Sustainability, Hong Kong University of Science and Technology, Hong Kong 999077, China; Department of Chemistry, Hong Kong University of Science and Technology, Hong Kong 999077, China; Division of Environment and Sustainability, Hong Kong University of Science and Technology, Hong Kong 999077, China; State Key Joint Laboratory of Environmental Simulation and Pollution Control, International Joint laboratory for Regional Pollution Control, College of Environmental Sciences and Engineering, Peking University, Beijing 100871, China; State Key Joint Laboratory of Environmental Simulation and Pollution Control, International Joint laboratory for Regional Pollution Control, College of Environmental Sciences and Engineering, Peking University, Beijing 100871, China; Frontier Science Center for Deep Ocean Multispheres and Earth System and Physical Oceanography Laboratory, Ocean University of China, Qingdao 266100, China; College of Oceanic and Atmospheric Sciences, Ocean University of China, Qingdao 266100, China; Shenzhen Key Laboratory of Precision Measurement and Early Warning Technology for Urban Environmental Health Risks, School of Environmental Science and Engineering, Southern University of Science and Technology, Shenzhen 518055, China; Guangdong Provincial Observation and Research Station for Coastal Atmosphere and Climate of the Greater Bay Area, Southern University of Science and Technology, Shenzhen 518055, China; Shenzhen Key Laboratory of Precision Measurement and Early Warning Technology for Urban Environmental Health Risks, School of Environmental Science and Engineering, Southern University of Science and Technology, Shenzhen 518055, China; Guangdong Provincial Observation and Research Station for Coastal Atmosphere and Climate of the Greater Bay Area, Southern University of Science and Technology, Shenzhen 518055, China; Shenzhen Key Laboratory of Precision Measurement and Early Warning Technology for Urban Environmental Health Risks, School of Environmental Science and Engineering, Southern University of Science and Technology, Shenzhen 518055, China; Guangdong Provincial Observation and Research Station for Coastal Atmosphere and Climate of the Greater Bay Area, Southern University of Science and Technology, Shenzhen 518055, China; Shenzhen Key Laboratory of Precision Measurement and Early Warning Technology for Urban Environmental Health Risks, School of Environmental Science and Engineering, Southern University of Science and Technology, Shenzhen 518055, China; Guangdong Provincial Observation and Research Station for Coastal Atmosphere and Climate of the Greater Bay Area, Southern University of Science and Technology, Shenzhen 518055, China; Department of Ocean Science, Hong Kong University of Science and Technology, Hong Kong 999077, China; Department of Ocean Science and Engineering, Southern University of Science and Technology, Shenzhen 518055, China

In the article by Li *et al., ‘*Dissecting the contributions of organic nitrogen aerosols to global atmospheric nitrogen deposition and implications for ecosystems*’, National Science Review* 2023; **10**: nwad244, https://doi.org/10.1093/nsr/nwad244, errors were identified concerning Fig. 4G, Table 1 and the corresponding values in the text.

The specific corrections are as follows:

1. **Global atmospheric burden of ON**: Our original calculation did not properly account for the vertical variation of air density, leading to an overestimation of the ON burden (Column 2 in Table 1). We correct by using the correct air density profile.
2. **Imine secondary organic nitrogen (SON) formation**: In the original version, we calculated imine SON formation assuming constant reactant abundance. We update this calculation by using pseudo-first-order rate constants, where ammonium (NH₄⁺) is assumed constant, while glyoxal and methylglyoxal decrease over timesteps.

**Figure 4.**
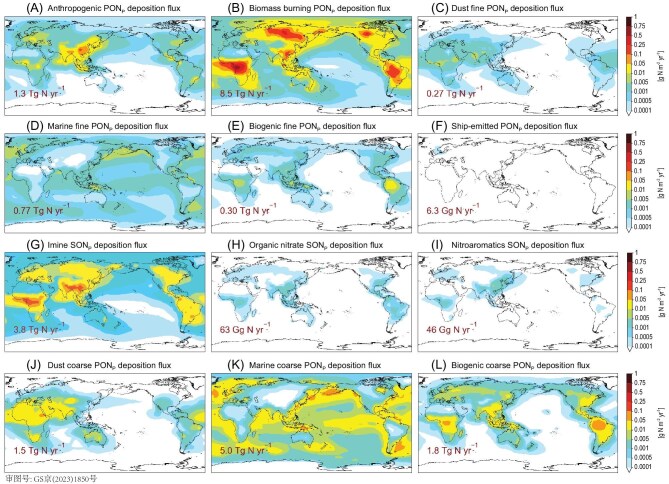
Simulated annual mean atmospheric deposition fluxes of ON_p_ from different sources. (A) anthropogenic PON_p_; (B) biomass burning PON_p_; (C) fine dust PON_p_; (D) fine marine PON_p_; (E) fine biogenic PON_p_; (F) ship-emitted PON_p_; **(G) imine SON_p_**; (H) organic nitrate SON_p_; (I) nitroaromatic SON_p_; (J) coarse dust PON_p_; (K) coarse marine PON_p_; (L) coarse biogenic PON_p_.

**Table 1.** Global budget of atmospheric ON and the ON : TN ratios in atmospheric deposition as simulated by the GEOS-Chem model.

**Source types**	**Atmospheric burden (New)** **[Tg N]**	**Emission** **[Tg N yr^−^^1^]**	**Net chemical production^a^** **[Tg N yr^−^^1^]**	**Dry deposition^b^** **[Tg N yr^−^^1^]**	**Wet deposition^b^** **[Tg N yr^−^^1^]**	**Total deposition^b^** **[Tg N yr^−^^1^]**	**Total deposition to the ocean^b^** **[Tg N yr^−^^1^]**
**Total ON (ON_p_ + ON_g_)**	**0.42**	20	5.8	6.0 (5.0)	20 (17)	26 (22)	11 (8.0)
**Particulate ON (ON_p_)**	**0.22**	19	3.9	4.0 (3.0)	19 (17)	23 (20)	10 (7.5)
***Fine mode* (ON_fp_)**							
Anthropogenic emissions	**0.015**	1.3	-	0.29 (0.23)	1.0 (1.0)	1.3 (1.2)	0.51 (0.47)
Biomass burning emissions	**0.11**	8.5	-	1.7 (1.4)	6.7 (6.5)	8.5 (7.9)	2.4 (2.3)
Dust emissions	**0.0055**	0.27	-	0.059 (0.042)	0.21 (0.20)	0.27 (0.24)	0.11 (0.10)
Primary biological particle emissions	**0.0056**	0.30	-	0.053 (0.053)	0.25 (0.25)	0.30 (0.30)	0.074 (0.074)
Ship emissions	**0.000050**	0.0063	-	0.0016 (0.0012)	0.0047 (0.0043)	0.0063 (0.0055)	0.0049 (0.0049)
Marine emissions	**0.0024**	0.77	-	0.22 (0.12)	0.55 (0.45)	0.77 (0.57)	0.65 (0.46)
Organic nitrate SON_p_	**0.00051**	-	0.069	0.028 (0.028)	0.040 (0.040)	0.068 (0.068)	0.016 (0.016)
NAC SON_p_	**0.00062**	-	0.046	0.011 (0.011)	0.035 (0.035)	0.046 (0.046)	0.015 (0.015)
Imine SON_p_	**0.043**	-	3.8	0.56 (0.56)	3.2 (3.2)	3.8 (3.8)	0.98 (0.98)
***Coarse mode* (ON_cp_)**							
Dust emissions	**0.017**	1.5	-	0.22 (0.14)	1.3 (1.0)	1.5 (1.1)	0.44 (0.40)
Primary biological particle emissions	**0.0044**	1.8	-	0.13 (0.13)	1.7 (1.7)	1.8 (1.8)	0.33 (0.33)
Marine emissions	**0.012**	5.0	-	0.63 (0.31)	4.4 (2.3)	5.0 (2.6)	4.6 (2.4)
**Gaseous ON (ON_g_)**	**0.2**	0.75	1.7	2.0 (2.0)	0.45 (0.41)	2.5 (2.5)	0.49 (0.49)
**ON:TN in deposited fluxes**	**-**	-	-	11% (10%)	28% (25%)	21% (18%)	23% (18%)

The corrected Table 1 and corresponding text are presented below. Corrections involve the values of atmospheric burden (second column from left in Table 1, and those in the main text; both highlighted in bold).

## GLOBAL BUDGET OF ATMOSPHERIC ON AND CONTRIBUTION TO ATMOSPHERIC TN DEPOSITION

Table 1 summarizes the global budget of atmospheric ON as simulated by our model. The total atmospheric burden of ON was **0.4** Tg N (range in sensitivity experiments was **0.26** Tg N to **0.56** Tg N), including **0.2** Tg N of ON_g_ and **0.2** Tg N of ON_p_. ON_g_ species were mostly chemically produced in the atmosphere as acyl peroxy nitrates (e.g. peroxyacetyl nitrate) and non-acyl peroxy nitrates (e.g. methyl peroxy nitrate), and all ON_g_ species had limited solubility [2]. As such, ON_g_ were mainly removed from the atmosphere by thermal decomposition, photolysis or OH oxidation [26,60], with deposition accounting for a mere 1% to 2% of its global sink [61]. Globally, ON_g_ only constituted 9% of the total atmospheric ON deposition. In contrast, ON_p_ constituted only **50%** of the global atmospheric ON burden but dominated the global atmospheric ON deposition (91%). Of the **0.2** Tg N global atmospheric ON_p_ burden, 87% (**0.18** Tg N) was in the fine mode (ON_fp_). ON_cp_ constituted only 13% (**0.03** Tg N) of the global ON_p_ burden because of its rapid deposition.

The corrected Fig. 4 is presented below. There is a slight difference in Fig. 4G while the other sub-figures remain unchanged.

The publicly accessible model code has been updated at the original repository link: https://doi.org/10.57760/sciencedb.o00005.00024

We apologize for any inconvenience caused. These corrections do not affect the conclusions of the article.

