# Supplementary material for: Dissecting the contributions of organic nitrogen aerosols to global atmospheric nitrogen deposition and implications for ecosystems
Source: Natl Sci Rev. 2023 Sep 18;10(12):nwad244. doi: 10.1093/nsr/nwad244 (PMC10634623; doi:10.1093/nsr/nwad244)
Supplement: nwad244_Supplemental_Files [file nwad244_supplemental_files.zip › SI for NSR 20230829.docx]

Supplementary Materials for

**Dissecting the contributions of organic nitrogen aerosols to global atmospheric nitrogen deposition and implications for ecosystems**

Yumin Li^1,2,3^, Tzung-May Fu^1,2,4^*, Jian Zhen Yu^3,5^*, Xu Yu^3^, Qi Chen^6^, Ruqian Miao^6^, Yang Zhou^7,8^, Aoxing Zhang^1,2^, Jianhuai Ye^1,2^, Xin Yang^1,2^, Shu Tao^1,2^, Hongbin Liu^9^, Weiqi Yao^10^

* Corresponding authors: Tzung-May Fu, Jian Zhen Yu

Email: [fuzm@sustech.edu.cn](mailto:fuzm@sustech.edu.cn); [chjianyu@ust.hk](mailto:chjianyu@ust.hk)

**This PDF file includes:**

Supplementary Texts S1 to S3

Figures S1 to S18

Tables S1 to S4

Legends for Datasets S1 to S3

**Other Supplementary Materials for this manuscript include the following:**

Datasets S1 to S3

Supplementary Texts

**Text S1: Supplementary details of materials and methods**

1. Global atmospheric ON simulation
2. Model framework

We developed a global atmospheric gaseous and particulate ON simulation for the year 2016 using the GEOS-Chem global 3-D chemical transport model (v12.9.3, <http://geos-chem.org>) [1]. GEOS-Chem was driven by the NASA GEOS-FP assimilated meteorological dataset (<https://gmao.gsfc.nasa.gov/GMAO_products/>), which had a native resolution of 0.3125^o^ longitude × 0.25^o^ latitude and 72 vertical layers. To drive GEOS-Chem, we downgraded the horizontal resolution to 5^o^ longitude × 4^o^ latitude. Narrative descriptions of the GEOS-Chem model and full model references can be found at <https://geos-chem.seas.harvard.edu/geos-chem-narrative>.

GEOS-Chem included a comprehensive tropospheric HO*_x_*-NO*_x_*-VOC-ozone-halogen-aerosol chemical mechanism. The standard aerosol scheme in GEOS-Chem described the atmospheric evolutions of primary elemental carbon aerosol, primary organic carbon aerosol (POC), natural and anthropogenic dust, sea salt, sulfate, nitrate, ammonium, and the carbonaceous component of secondary organic aerosol (SOA). Secondary inorganic aerosol production was calculated with the ISORROPIA II (version 2.2) [2] thermodynamic equilibrium module. All freshly emitted OC was assumed to be 50% hydrophobic, with a conversion timescale of 1.2 days from hydrophobic to hydrophilic. SOA was produced at prescribed yields from the irreversible oxidations of gaseous precursors from biomass burning, anthropogenic, and biogenic sources. The organic aerosol to organic carbon mass ratio was assumed to be 1.4 for primary organic aerosol and 2.1 for SOA. The dry depositions of gases and aerosols were represented with a resistance-in-series scheme. The aerodynamic resistances and maximum deposition velocity for aerosols vary based on the type of land cover. Aerosols and soluble gases were wet-scavenged via washout and rainout processes. We spun up the model through the year 2016 and used the results to restart the simulation on January 1, 2016.

Global monthly anthropogenic emissions of OC and other precursors for the year 2016 were from the Community Emissions Data System (CEDS, native resolution 0.3125° longitude × 0.25° latitude, <https://esgf-node.llnl.gov/search/input4mips/>) but superseded by the following regional inventories: the National Emissions Inventory 2011 over the U.S. (NEI11, <http://ww.epa.gov/air-emissions-inventories>), the Air Pollutants Emissions Inventory over Canada (APEI, <http://ec.gc.ca/inrp-npri/donnees-data/ap>), the MIX Inventory over East Asia (<http://meicmodel.org>), and the Diffuse and Inefficient Combustion Emissions in Africa inventory over Africa (DICE-Africa).

Global monthly biomass burning emissions were from the Global Fire Emissions Database including small fires (GFEDv4.1s, native resolution 0.25^o^, <http://www.globalfiredata.org>). Biogenic VOC emissions were simulated online in GEOS-Chem using the MEGAN (v2.1) algorithm. Natural dust was emitted in four size bins. Dust mass in the fine particulate mode (≤2.5 μm in diameter) was represented by 100% of the dust mass emitted into the first dust bin (0.1 μm to 1.0 μm in radii) and 38% of the dust mass emitted into the second dust bin (1.0 μm to 1.8 μm in radii). The remaining dust mass was allocated to the coarse particulate mode (>2.5 μm in diameter). Anthropogenic dust emissions were assumed to be entirely in the fine mode. Sea salt emissions in the accumulation (0.1 μm to 0.5 μm in radii) and coarse modes were calculated online in GEOS-Chem.

We added to GEOS-Chem the fine and coarse POC emitted as part of sea sprays using a scheme dependent on wind speeds and the chlorophyll-a concentrations in the surface ocean. Aerosolized fine POC from sea sprays were mostly insoluble colloids consisting of lipopolysaccharides, which we assumed to be 100% hydrophobic. Coarse POC aerosolized from sea sprays contained mixtures of organic carbon in sea salts and biological debris, which we assumed to be 20% hydrophilic. Emissions of fine and coarse POC associated with natural and anthropogenic dust were estimated by scaling dust emissions with the measured organic matter (OM) content of soil (0.9%) and an assumed OM to OC mass ratio of 1.7. Freshly emitted OC in natural dust was assumed to be 90% hydrophobic based on observations. Freshly emitted OC in anthropogenic dust was assumed to be 50% hydrophobic, the same as freshly emitted anthropogenic OC. We also included in GEOS-Chem additional fine and coarse POC as part of the terrestrial primary biological aerosol particles (PBAPs). We assumed PBAPs to be 100% soluble, 25% of their mass were in the fine mode, and their OM-to-OC mass ratio was 2.6 [3].

1. Primary emissions of atmospheric particulate and gaseous ON

To estimate the global emissions of primary particulate ON (PON_p_) from different sources, we scaled the primary particulate OC emissions using source-specific N:C mass ratios from the literature [4-6] and our measurements [7] (Table S1). We assumed that all PON_p_ associated with anthropogenic combustions, anthropogenic dust, and biomass burning were in the fine mode only, while PON_p_ associated with natural dust, PBAPs, and sea sprays were in both the fine and coarse modes. In contrast to previous simulations [4, 5] that assumed all combustion sources had the same N:C ratio, we differentiated between biomass burning and anthropogenic combustion emissions as distinct sources with source-specific N:C ratios. We began with the mean N:C mass ratio (Table S1) reported in the literature [4] for fine ON_p_ emitted from biomass burning, dust, and sea sprays, as well as for coarse ON_p_ emitted as part of dust, PBAPs, and sea sprays. For fine ON_p_ emitted from anthropogenic combustion sources (power generation, industry, residential sources, land transportation, biofuel use, and ships), we used the updated mean N:C mass ratios from our own measurements, because these measurements sampled sites representing typical urban sources. Also, our measurements were conducted using new thermal techniques that directly quantified ambient ON_p_ concentrations, instead of deriving ON_p_ concentrations by subtracting the inorganic nitrogen (IN) components from the total nitrogen (TN) abundance [7]. Our N:C mass ratios for these sources were one-third to one-sixth of the values reported in previous literature. Then, we conducted trial simulations for global atmospheric ON_p_ concentrations and deposition fluxes to estimate the uncertainty resulting from different N:C ratios in various ON sources. In the end, for fine ON_p_ emitted from biomass burning, we used an N:C value (Table S1) on the high end of the values reported in the literature, because the use of that high-end N:C value (0.6) improved the agreement of our simulated ON deposition fluxes against measurements, while also reproducing the observed surface ON_p_ concentrations at global sites. For coarse ON_p_ emitted as parts of dust, PBAPs, and sea sprays, we used the maximum N:C mass ratios from the ranges reported in the literature, because the use of these maximum N:C ratios improved the agreement of our simulated ON deposition fluxes against measurements while also reproducing the observed surface ON_p_ concentrations.

Our choice of N:C mass ratios for the standard simulation was later justified by our further sensitivity experiments, in which we found that increasing the anthropogenic PON_p_ emissions by a factor of 9 would result in good agreement between the simulated and observed ON deposition fluxes but would lead to an overestimation of surface ON_fp_ abundance by a factor of 4. In contrast, increasing biomass burning PON_p_ emissions or imine SON_p_ production by a factor of 5, respectively, would both result in good agreements between the simulated and observed ON deposition fluxes, while the simulated surface ON_fp_ concentrations would only be larger than current observations by a factor of 2. These findings showed that even with a relatively high N:C mass ratio in our standard simulation, our simulated ON_p_ from biomass burning may be biased low, and that further measurements representing the biomass burning ON_p_ emissions are need to better constrain the global abundance and deposition fluxes of ON.

We assumed that freshly emitted ON_p_ has the same hygroscopic properties as the freshly emitted OC from that specific source (Table S1). Finally, we assumed that primary hydrophobic ON_p_ becomes hydrophilic on a timescale of 1.2 days, consistent with the aging of POC in GEOS-Chem model.

For primary sources of gaseous ON (ON_g_), we included the low-molecular-weight (≤C_6_) amines emitted from anthropogenic (particularly agricultural) activities, biomass burning, and marine sources [8]. Regional estimates indicated that the emissions of amines from these sources are 2 to 3 orders of magnitude smaller than the corresponding emissions of ammonia [8]. We thus scaled the global emissions of ammonia from these sources by 1% to represent global amine emissions.

1. Formation pathways of secondary ON in the atmosphere

We included three major pathways of secondary particulate ON (SON_p_) formation in GEOS-Chem based on previous laboratory studies (Fig. 1): (1) the gas-phase oxidation of aliphatic VOCs in the presence of NO_x_ to form semi-volatile organic nitrates, which are then irreversibly up-taken at the surface of aqueous particles; (2) the gas-phase oxidation of aromatic VOCs in the presence of NO_x_ to form semi-volatile nitroaromatics, which partition into the particle phase; and (3) the aqueous reactions of dicarbonyls with ammonium or amines in cloud droplets and wet aerosols to from heterocyclic compounds with imine or amine functional groups. The first two pathways produced oxidized SON_p_, while the third pathway produced reduced SON_p_. The model representations of these formation pathways are detailed showing below.

c1 Formation of secondary particulate organic nitrate (oxidized ON)

Organic nitrates (RONO_2_) are formed via the oxidation of non-methane volatile organic compounds (NMVOCs) in the presence of reactive N. During the day, OH-oxidation of NMVOCs produces peroxy radicals (RO_2_), which may react with NO to form RONO_2_. At night, NMVOCs may be oxidized by NO_3_ to form RO_2_ (RONO_2_-RO_2_), which then react with HO_2_, RO_2_, or NO_3_ to generate stable aldehyde- or alcohol-nitrate products (Fig. 1) [9-13]. Of all the secondary organic nitrates produced by various NMVOC precursors, only those produced by the oxidation of isoprene and monoterpenes have been observed in the particulate phase [13, 14]. Following a previous model study [10], we simulated the uptake of these biogenic gaseous organic nitrates by wet aerosol surfaces (RH ≥ 35%) using uptake coefficients of γ=0.005 for isoprene nitrates and γ=0.01 for all monoterpene nitrates. Particulate RONO_2_ were removed by dry and wet deposition. We also assumed that all particulate RONO_2_ were removed by hydrolysis to form HNO_3_ at a timescale of 1 hour [10]. Gas-phase RONO_2_ products from all NMVOC precursors were also removed by OH oxidation, photolysis, and deposition [10, 11, 15].

c2 Formation of secondary nitroaromatic compounds (oxidized ON)

Secondary nitroaromatic compounds (NACs, here including nitrophenol, nitrocatechol, methyl-nitrophenol, methyl-nitrocatechol, dimethyl-nitrophenol, and dimethyl-nitrocatechol) form when aromatic-OH adducts are oxidized by OH or NO_3_ radicals in the presence of NO_2_ (Fig. 1) [16, 17]. We considered both primary and secondary sources of aromatic-OH adducts in our model. Phenol is emitted from biomass and biofuel burning. We calculated the global phenol emissions using the GFED4.1s inventory, the CEDS inventory (superseded by the DICE-Africa inventory over Africa), and published emission factors [18]. Secondary aromatic-OH adducts in the model included phenol, catechol, cresol, methyl-catechol, xylenol, and dimethyl-catechol, which were produced by the OH-oxidations of benzene, toluene, and xylenes [19]. The gas-phase reactions from aromatic hydrocarbons to from aromatic-OH adducts and the subsequent gas-phase reactions to form NACs were extracted from the Master Chemical Mechanism (MCMv3.3.1, accessible at  [http://mcm.york.ac.uk)](http://mcm.leeds.ac.uk/MCM/)) [20].

Observations showed that more than 95% of the ambient methyl-nitrocatechol, dimethyl- nitrophenol, and dimethyl-nitrocatechol are in the particle phase [17, 21]. We thus assumed these products to be non-volatile which immediately condense into the particulate phase upon formation. Other NACs species (nitrophenol, nitrocatechol, methyl-nitrophenol) were assumed to be semi-volatile; their gas-particle partitioning was calculated using gas-particle partitioning equilibrium constants $K_{\mathrm{OM},i}$ (unit: m^3^ μg^−1^) [17]:

$K_{\mathrm{OM},i}= \frac{A_{i}}{G_{i}M_{\mathrm{OM}}}=\frac{RT}{{10}^{6}MW_{\mathrm{OM}}\zeta_{i}P_{i}}$ (S1)

where $G_{i}$ (unit: μg m^−3^) and $A_{i}$ (unit: μg m^−3^) were the concentrations of species $i$ in the gas and particulate phases, respectively. $M_{\mathrm{OM}}$ (unit: μg m^−3^) was the mass concentration of the organic aerosol substrate onto which the semi-volatile species may condense. $R$ was the dry air gas constant (8.206 × 10^-5^ m^3^ atm mol^-1^ K^-1^), *T* (unit: K) was the temperature, $MW_{\mathrm{OM}}$ (200 g mol^−1^) was the mean molecular weight of the organic aerosol substrate. $\zeta_{i}$ was the activity coefficient of organic matter, assumed to be 1. $P_{i}$ (unit: atm) was the saturation vapor pressure of species $i$ as a pure liquid at 298 K. Table S2 shows the $P_{i}$ of nitrophenol, nitrocatechol, and methyl-nitrophenol used in this study. These $P_{i}$ values were averages of theoretical estimates [17] and measured values [21]. However, the particulate fractions of nitrophenol (0.2%-1%) and methyl-nitrophenol (3%-5%) calculated from Eq. S1 was considerably lower than those observed. Limited ambient observations suggested that the particulate fractions ranged between 17% and 82% for nitrophenol, and between 26% and 78% for methyl-nitrophenol (Dataset S2) [16, 17, 22]. Observed particulate fractions for both compounds showed considerable variability, possibly due to volatility differences among isomers or due to unknown measurement errors [16, 17, 22]. To match the observed particulate fractions of these NACs species, we manually increased the $K_{OM,i}$ calculated from Eq. S1 by 20 times for nitrophenol and by 10 times for methyl-nitrophenol, respectively.

c3 Formation of imine SON production via aqueous reactions of dicarbonyls (reduced ON)

Glyoxal and methylglyoxal in the atmosphere are emitted from biomass and biofuel burning, as well as produced from the photooxidation of many NMVOCs [23]. Laboratory studies have found that glyoxal and methylglyoxal can react with ammonium (NH_4_^+^), amines, and amino acids in the aqueous phase to produce reduced SON compounds, such as imines, diimines, imidazoles, and N-containing oligomers [24-29]. Of all these aqueous reactions of dicarbonyls, the reactions with ammonium appeared to be the dominant reduced SON formation pathway [24, 28]. In experiments where the dicarbonyls reacted with ammonium, amines, and amino acids in the aqueous phase at pH ranging from 5 to 6, the reactions with ammonium contributed 67%-99% (glyoxal) and 69%-86% (methylglyoxal) of the total SON formation, respectively [28]. Also, measurements showed that the aqueous reaction rates of dicarbonyls with amines and amino acids were 2 to 3 orders of magnitudes lower than the reactions rates of dicarbonyls with ammonium at pH=5.5 and under typical concentrations in the atmosphere [28]. Therefore, we simulated only the aqueous reactions of dicarbonyls with ammonium to form SON in cloud droplets and wet aerosols. Glyoxal and methylglyoxal also reacted with OH radicals in cloud droplets and wet aerosols to form non-N SOA in our model [9, 30].

For cloud droplets, the cloud water composition was computed locally in each grid cell containing liquid cloud water over 30 min time steps using the in-cloud liquid water content and cloud volume fraction from NASA GEOS-FP assimilated meteorological dataset [31]. The in-cloud acidity was determined by the balance between dissolved acids and bases [31, 32]. The dissolutions of glyoxal and methylglyoxal into the cloud water were simulated using their temperature-dependent effective Henry’s law constants (units: M atm^-1^):

$K_{\mathrm{Gly}}=4.15 \times{10}^{5} \cdot\text{exp}\left[ 7200 \cdot\left( \frac{1}{T}-\frac{1}{298.15} \right) \right]$ (S2)

$K_{\mathrm{MG}}=3.24 \times{10}^{4} \cdot\exp\left[ 6200 \cdot\left( \frac{1}{T}-\frac{1}{298.15} \right) \right]$ (S3)

The ammonium ([NH_4_^+^]_aq_) in cloud water originated from either the dissolution of gaseous ammonia or the scavenging of particulate ammonium by cloud droplets. GEOS-Chem calculated the thermodynamic equilibrium between ammonia and ammonium using the ISOPROPIA II algorithm. Gaseous ammonia partition into the aqueous phase according to its effective Henry’s law constant ($K_{NH_{3}}^{*}$, unit: M atm^−1^), which was temperature- and pH-dependent [30]:

$K_{NH_{3}}^{*}=K_{NH_{3}}\cdot\left[ 1+(\frac{K_{a}*pH_{\mathrm{cloud}}}{K_{w}}) \right]$ (S4)

where $K_{NH_{3}}=60 \cdot\text{exp}\left[ 4200 \cdot\left( \frac{1}{T}-\frac{1}{298.15} \right) \right]$ , $K_{a}=1.7 \times{10}^{-5}\cdot\text{exp}\left[ -450 \cdot\left( \frac{1}{T}-\frac{1}{298.15} \right) \right]$, and $K_{w}= 1\times{10}^{-14} \cdot\text{exp}\left[ -6710 \cdot\left( \frac{1}{T}-\frac{1}{298.15} \right) \right].$ In Eq. S4 pH_cloud_ was the pH value of cloud water. We assumed a cloud scavenging efficiency of 0.7 for fine-mode aerosols, which expressed the effective probability of aerosol mass being scavenged upon collision with cloud droplets [31, 32].

The reaction orders of dicarbonyls with ammonium in the aqueous phase were dependent on the abundances of reactants [24, 26, 28]. For glyoxal, laboratory measurements showed that the reaction kinetics transitioned from second-order to fourth-order at [Gly]*_aq_* × [NH_4_^+^]*_aq_*≥1.2 M^2^ [24, 26, 28]. For methylglyoxal, the reaction kinetics was also concentration-dependent [25], although a transition threshold had not been identified. For typical in-cloud conditions, the [Gly]*_aq_* × [NH_4_^+^]*_aq_* is always lower than the 1.2M^2^ threshold. We therefore assumed the in-cloud reactions between glyoxal and ammonium to be second-order and applied the same assumption to methylglyoxal. The in-cloud reactions of dicarbonyls with ammonium form intermediate products, such as imines and diimines, which are relatively unstable [27, 28]. However, because formaldehyde or acetaldehyde is always present in the cloud water, diimines may act as nucleophiles in attacking the carbonyl group of formaldehyde or acetaldehyde to form acyclic enol intermediates, which then go on to close their ring and form more stable, non-volatile imidazoles and nitrogen-containing oligomers [27]. As a result, the effective production rates of non-volatile ON by the aqueous reactions of dicarbonyls with ammonium in the presence of formaldehyde and acetaldehyde are 1.8 times the rates in pure water [33]. The effective in-cloud reaction rates of glyoxal ($r_{\mathrm{Gly}, c}$, unit: M s^-1^) and methylglyoxal ($r_{\mathrm{MG}, c}$, unit: M s^-1^) with ammonium are described in Eqs. S5 and S6:

$r_{Gly, c}=\left[ \mathrm{Gly} \right]_{c}\cdot\left[ NH_{4}^{+} \right]_{c}\cdot k_{Gly, \mathrm{NH}_{4}^{+}}^{\mathrm{II}}\cdot1.8$ (S5)

$r_{MG, c}=\left[ \mathrm{MG} \right]_{c}\cdot\left[ NH_{4}^{+} \right]_{c}\cdot k_{MG, \mathrm{NH}_{4}^{+}}^{\mathrm{II}}\cdot1.8$ (S6)

where [Gly]*_c_*, [MG]*_c_*, [NH_4_^+^]*_c_* were the reactant concentrations in the cloud water. The pH-dependent bimolecular rate constants [28] were $k_{Gly, \mathrm{NH}_{4}^{+}}^{\mathrm{II}}= {10}^{\left( 1.05\pm0.03 \right) pH_{cloud}-7.45}$ (unit:M^-1^ s^-1^), $k_{MG, \mathrm{NH}_{4}^{+}}^{\mathrm{II}}={10}^{\left( 0.834\pm0.2 \right) pH_{cloud}-5.91}$ (unit: M^-1^ s^-1^). The aqueous reactions of glyoxal and methylglyoxal with OH in cloud droplets to form non-N SOA were parameterized as irreversible uptake processes [30]. The resulting SON product and non-N SOA both remained in the particulate phase upon cloud evaporation [29].

The solubilities of glyoxal and methylglyoxal in wet aerosols were affected by the salt contents of the particles. Laboratory studies found that, with increasing salt concentrations, glyoxal became more soluble (“salts in”), while methylglyoxal became less soluble (“salts out”). The effective Henry’s law constants of dicarbonyls ($K_{salt, i}^{*}$; unit: M atm^−1^) in salt-containing wet aerosols were [30]:

$\log\left( \frac{K_{i}}{K_{salt, i}^{*}} \right)=K_{s,\left( \mathrm{NH}_{4} \right)_{2}\mathrm{SO}_{4}}\left[ \left( \mathrm{NH}_{4} \right)_{2}\mathrm{SO}_{4} \right]_{aq,a}+K_{s,{\mathrm{NH}_{4}\mathrm{NO}}_{3}}\left[ {\mathrm{NH}_{4}\mathrm{NO}}_{3} \right]_{aq,a}$ (S7)

where $K_{i}$ was the Henry’s law constant of dicarbonyls in pure water. $\left[ \left( \mathrm{NH}_{4} \right)_{2}\mathrm{SO}_{4} \right]_{aq,a}$ and $\left[ {\mathrm{NH}_{4}\mathrm{NO}}_{3} \right]_{aq,a}$ were the molal concentrations of ammonium sulfate and ammonium nitrate in wet aerosols. $K_{s,\left( \mathrm{NH}_{4} \right)_{2}\mathrm{SO}_{4}}$ and $K_{s,{\mathrm{NH}_{4}\mathrm{NO}}_{3}}$ were the salting constants (Table S3).

In wet aerosols, the reaction pathways of dicarbonyls with ammonium were similar to those in cloud water, except that the concentrations of dicarbonyls and ammonium were higher. As such, for glyoxal both second-order and fourth-order reactions may take place in wet aerosols, depending on the reactant abundances ([Gly]*_aq_* × [NH_4_^+^]*_aq_*) [24, 26, 28]. For methylglyoxal, only the second-order reaction rate constants have been measured, so we assumed that the reaction of methylglyoxal with ammonium proceeds as a second-order reaction in wet aerosols. In addition, the reaction rates of dicarbonyls with ammonium in wet aerosols were dependent on RH and the ion activity of ammonium (assumed to be 0.6 based on laboratory measurements) [24]. The wet aerosol reaction rates of glyoxal ($r_{\mathrm{Gly}, a}$, unit: M s^-1^) and methylglyoxal ($r_{\mathrm{MG}, a}$, unit: M s^-1^) with ammonium were:

$r_{\mathrm{Gly}, a}= \alpha_{\mathrm{Gly}}\cdot0.6\left[ NH_{4}^{+} \right]_{aq,a}\cdot\left[ \mathrm{Gly} \right]_{aq, a}\cdot k_{Gly, \mathrm{NH}_{4}^{+}}^{\mathrm{II}}$ , for [NH_4_^+^]*_aq,a_* ∙ [Gly]*_aq,a_* < 1.2M^2^ (S8a)

$r_{\mathrm{Gly}, a}= \alpha_{\mathrm{Gly}}\cdot\left( 0.6\left[ NH_{4}^{+} \right]_{aq, a} \right)^{2}\cdot{\left[ \mathrm{Gly} \right]_{aq, a}}^{2}\cdot k_{Gly, \mathrm{NH}_{4}^{+}}^{\mathrm{IV}}$ , for [NH_4_^+^]*_aq,a_* ∙ [Gly]*_aq,a_* ≥ 1.2M^2^  (S8b)

$r_{\mathrm{MG}, a}= \alpha_{\mathrm{MG}}\cdot0.6\left[ NH_{4}^{+} \right]_{aq, a}\cdot\left[ \mathrm{MG} \right]_{aq, a}\cdot k_{MG, \mathrm{NH}_{4}^{+}}^{\mathrm{II}}$ (S9)

where [Gly]*_aq,a_*, [MG]*_aq,a_*, [NH_4_^+^]*_aq,a_* were the reactant concentrations in the wet aerosol. The pH-dependent 4^th^ order reaction constant for glyoxal [26] was $k_{Gly, \mathrm{NH}_{4}^{+}}^{\mathrm{IV}}= 1.43\pm0.25\times{10}^{-11}\cdot{10}^{pH}$(unit: M^-3^ s^-1^). $\alpha_{\mathrm{Gly}}$ and $\alpha_{\mathrm{MG}}$ were the RH acceleration coefficients for glyoxal and methylglyoxal, respectively (Table S3) [34].

We represented the total irreversible uptake of glyoxal and methylglyoxal by aqueous aerosols to form SOA (including the reaction with OH to form non-N SOA, and the reaction with ammonium to form SON) by parameterizing a reactive uptake coefficient ($\gamma_{i}$) for dicarbonyl species *i* (*i* is Gly or MG) [30]:

$\frac{1}{\gamma_{i}}=\frac{1}{\alpha}+ \frac{\omega_{i}}{4K_{i}^{*}RT\sqrt{k_{i, total}^{I}D_{aq}}}(\frac{1}{cothq-1/q})$ (S10)

where $\alpha=0.02$ was the mass accommodation coefficient, assumed to be identical to the $\alpha$ for formaldehyde uptake to water [30]. $\omega_{i}$ was the gas-phase thermal velocity of species *i*; $K_{i}^{*}$ was the effective henry’s law constant of species *i*; $R=0.08257$ was the universal gas constant; T (unit: K) was temperature. The assumed diffusion coefficient $D_{aq}={10}^{-9}$ (unit: m^2^ s^-1^), typical for small organic species. $q= {R_{p}}/l$ was the ratio of the wet aerosol radius $R_{p}$, to the diffusoreactive length for organic species $l=\left( \frac{D_{aq}}{k_{i}^{I}} \right)^{1/2}$ . The bulk loss rate constant of the dicarbonyls species *i* in the aqueous phase ($k_{i, total}^{I}$ , unit: M s^-1^) was the sum of the rates with ammonium and OH radical ($k_{i, total}^{I}=k_{i,\mathrm{NH}_{4}^{+}}^{I} + k_{i,\mathrm{OH}}^{I}$). We parameterized the SON production rates (Eqs. S8 and S9) as pseudo-first-order reactions for glyoxal ($r_{\mathrm{Gly}, a}= k_{Gly, \mathrm{NH}_{4}^{+}}^{I}\cdot\left[ \mathrm{Gly} \right]_{aq, a}$) and methylglyoxal ($r_{\mathrm{MG}, a}= k_{MG, \mathrm{NH}_{4}^{+}}^{I}\cdot\left[ \mathrm{MG} \right]_{aq, a}$), respectively. The pseudo-first-order rate constants for glyoxal ($k_{Gly, \mathrm{NH}_{4}^{+}}^{I}$) and methylglyoxal ($k_{MG, \mathrm{NH}_{4}^{+}}^{I}$) reacting with ammonium in a wet aerosol can be expressed as:

$k_{Gly, \mathrm{NH}_{4}^{+}}^{I}=\alpha_{\mathrm{Gly}}\cdot0.6\left[ NH_{4}^{+} \right]_{aq} \cdot k_{Gly, \mathrm{NH}_{4}^{+}}^{\mathrm{II}}$ , for [NH_4_^+^]*_aq,a_* ∙ [Gly]*_aq,a_* < 1.2M^2^ (S11a)

$k_{Gly, \mathrm{NH}_{4}^{+}}^{I}=\alpha_{\mathrm{Gly}}\cdot\left( 0.6\left[ NH_{4}^{+} \right]_{aq} \right)^{2}\cdot\left[ \mathrm{Gly} \right]_{aq, a}\cdot k_{Gly, \mathrm{NH}_{4}^{+}}^{\mathrm{IV}}$ , for [NH_4_^+^]*_aq,a_* ∙ [Gly]*_aq,a_* ≥1.2M^2^  (S11b)

$k_{MG, \mathrm{NH}_{4}^{+}}^{I}= \alpha_{\mathrm{MG}}\cdot0.6\left[ NH_{4}^{+} \right]_{aq}\cdot k_{MG, \mathrm{NH}_{4}^{+}}^{\mathrm{II}}$ (S12)

The pseudo-first-order order reaction rates for glyoxal and methylglyoxal reacting with OH radical were calculated from OH radical concentrations and the bimolecular rate constants.[30] The mole fraction of SON in the total SOA produced from the irreversible uptake described in Eq. S10 was calculated by the ratio between $k_{i,\mathrm{NH}_{4}^{+}}^{I}$ and $k_{i, total}^{I}$.

1. Chemical aging of gaseous and particulate ON

Gaseous ON species in our model included acyl peroxy nitrates (e.g., peroxyacetyl nitrate), non-acyl peroxy nitrates (e.g., methyl peroxy nitrate), gaseous organic nitrates, and gaseous amines. Atmospheric acyl peroxy nitrates were chemically removed by photolysis, thermal decomposition, and OH oxidation [35]. Atmospheric non-acyl peroxy nitrates were chemically removed by thermal decomposition and photolysis [36] (details and full references given in the narrative description of the GEOS-Chem model setting, <https://geos-chem.seas.harvard.edu/geos-chem-narrative>). Gaseous organic nitrates were mainly removed by photolysis and partition to the particle phase [10]. We included the gaseous oxidation of amines by OH radicals with the reaction rate constant $k=\left( 3.58 \pm0.22 \right)\times{10}^{-11}$ [unit: cm^3^ molecule^-1^ s^-1^], which resulted in global average lifetimes of gaseous amines on the order of hours [8].

There is currently limited understanding of the chemical fates of primary or secondary ON_p_ in the atmosphere. Field and laboratory measurements showed that the optical and hygroscopic properties of nitrogen-containing organic aerosol (OA) change with its age [37], possibly indicating structural changes in the ON molecules [38, 39]. Chamber experiments examined the optical properties of brown carbon aerosol produced from the photo-oxidation of aromatics in the presence of NO_x_ [40, 41]. Those studies showed that the mass absorption coefficient of the brown carbon aerosol decreased significantly when exposed to UV-radiation (i.e., photo-bleaching), but the ON: OC mass ratios in the brown carbon aerosol remained relatively stable or decreased only slightly [40, 41]. Field measurements also showed that the water-soluble ON (WSON) to water-soluble TN mass ratios of OA in polluted air masses increased with the age of the air masses, reflecting the continuous secondary ON production in the air masses and the relatively slow chemical removal of particulate ON content [42]. We thus assumed that there was no chemical degradation to remove the particulate ON content in primary or secondary ON_p_, except the hydrolysis of particulate organic nitrates [10]. We assumed that all freshly emitted hydrophobic ON converted to hydrophilic ON on a timescale of 1.2 days.

1. Observations of atmospheric ON_p_ concentrations and atmospheric ON deposition fluxes

Dataset S1 (references within) summarizes the measured concentrations of atmospheric ON_p_ and WSON_p_ concentrations compiled from the literature and our measurements [43] Most early measurements inferred ON_p_ (or WSON_p_) by subtracting the measured particulate IN from the particulate TN (details in Dataset S1 and S3) [44]. Only one study directly measured ON_p_ concentrations using thermal evolution and chemiluminescence detection techniques [43]. In all, ON_p_ and WSON_p_ measurements were made at 18 global surface sites (7 urban, 2 rural, 2 forested, 3 coastal, and 4 marine sites) and 62 global surface sites (15 urban, 6 rural, 8 forested, 11 coastal, and 22 marine sites), respectively. These measurements were mostly made for fine particulate matter samples (PM_1_ or PM_2.5_, 57 sites), but a small number of measurements sampled PM_10_ and total suspended particles (TSP). For comparison with our simulated fine ON_p_ concentrations, we used the measurements of fine particulate samples, except at some remote marine or forest sites where only samples of PM_10_ or TSP were available. We also assumed that measured WSON is the main component of ON in fine particles [44], given that most samples were of aged air masses and that aging tends to make N-containing OA more hydrophilic [37]. At sites where ON and WSON concentrations were measured for one year or longer, we compared the observed annual mean concentrations with the simulated annual mean concentration. At sites where measurements were made for less than one year, we compared the measured monthly mean concentrations at that site with the simulated monthly mean concentrations.

Dataset S2 (references within) summarizes the particulate NACs concentrations measured at 21 global surface sites, as well as the particulate organic nitrate concentrations measured at 30 global surface sites reported in the literature. For particulate NACs, most measurements sampled PM_2.5_; only two measurements sampled PM_10_ and one measurement sampled TSP. For particulate organic nitrate, all measurements sampled either PM_1_ or PM_2.5_. We compared our model results to all published measurements but note that these sampling differences may affect the comparison between simulation and observation.

Dataset S3 (references within) summarized the measured atmospheric ON deposition flux in throughfall and bulk precipitation samples, as well as the measured ON:TN ratio in the deposition samples reported in the literature. Most measurements in Dataset S3 analyzed only the dissolved part of N, i.e., dissolved ON (DON), dissolved IN (DIN), and dissolved total N (DTN= DIN + DON). The compilation of DON deposition measurements before 2011 was taken from a previous compilation [5], to which we supplemented with reports after 2011. Some studies only measured DON deposition flux in bulk precipitation samples (i.e., only measured wet deposition). We assumed that these wet deposition fluxes of precipitation samples were representative of the total deposition fluxes because atmospheric ON was removed mainly by wet deposition. However, the ON:TN ratios measured in precipitation samples may be higher than the ON:TN ratios in total N deposition because TN can also be removed by dry deposition. We compared the measured annual mean total ON deposition fluxes and ON:TN ratios with our simulated results.

Our compiled measurements spanned between 1999 and 2020 (Datasets S1, S2, S3). Based on the limited observations conducted at the same site in various years, we found that the interannual variation of observed ON_p_ concentrations or deposition fluxes may be a factor of 2 to 7. This uncertainty arising from the interannual variation of observed ON_p_ abundance and deposition fluxes was less than an order of magnitude and therefore less than the potential discrepancies between our simulated results and observations. It was thus reasonable to compare our simulated ON_p_ concentrations and deposition fluxes in 2016 with observations during 1999 to 2020, as interannual variation of observations was not the dominant cause of discrepancy between simulated results and observations.

**Text S2:** **Sensitivity simulations for** **global atmospheric ON**

The major uncertainty in our simulated ON_p_ concentration and deposition flux was associated with the source-specific N:C mass ratios for PON_p_ (Table S1) and the SON_p_ production rates. We conducted sensitivity simulations to evaluate the impacts of these model parameters on the simulated ON_p_ concentrations and deposition fluxes. We conducted (1) an upper-limit sensitivity simulation using the highest reported N:C mass ratios for PON_p_ sources and the fastest imine SON_p_ production rate, (2) and a lower-limit sensitivity simulation using the lowest reported N:C mass ratios for PON_p_ sources and the slowest imine SON_p_ production rate, We also conducted (3) a PON_p_-enhanced simulation using the highest reported N:C mass ratios for PON_p_ sources and the lowest imine SON_p_ production rate, and (4) a SON_p_-enhanced simulation using the lowest reported N:C mass ratios for PON_p_ sources and the fastest imine SON_p_ production rate. We did not perturb the production rates of organic nitrates SON_p_ and nitroaromatics SON_p_, as they each contributed less than 0.001 μg N m^-3^ (0.5%) of the simulated global mean surface ON_fp_ and less than 0.5% of the simulated global ON_p_ deposition in our standard simulation. Additionally, we analyzed the contributions from different ON_p_ sources (most important biomass burning, anthropogenic combustion, marine emission, and imine SON generation) to the overall simulated ON_fp_ concentrations on a site-by-site basis (Fig. S1).

For the imine SON_p_ production, the pH-dependent bimolecular rate constants [28] were $k_{Gly, \mathrm{NH}_{4}^{+}}^{\mathrm{II}}= {10}^{\left( 1.05\pm0.03 \right) pH_{cloud}-7.45}$ (unit:M^-1^ s^-1^), $k_{MG, \mathrm{NH}_{4}^{+}}^{\mathrm{II}}={10}^{\left( 0.834\pm0.2 \right) pH_{cloud}-5.91}$ (unit: M^-1^ s^-1^), the pH-dependent 4^th^ order reaction constant for glyoxal [26] was $k_{Gly, \mathrm{NH}_{4}^{+}}^{\mathrm{IV}}= 1.43\pm0.25\times{10}^{-11}\cdot{10}^{pH}$(unit: M^-3^ s^-1^). In our upper-limit SON_p_ generation rate sensitivity simulation, we used $k_{Gly, \mathrm{NH}_{4}^{+}}^{\mathrm{II}}= {10}^{\left( 1.05+0.03 \right) pH_{cloud}-7.45}$ (unit:M^-1^ s^-1^), $k_{MG, \mathrm{NH}_{4}^{+}}^{\mathrm{II}}={10}^{\left( 0.834+0.2 \right) pH_{cloud}-5.91}$ (unit: M^-1^ s^-1^), and $k_{Gly, \mathrm{NH}_{4}^{+}}^{\mathrm{IV}}= 1.43+0.25\times{10}^{-11}\cdot{10}^{pH}$. In the lower-limit SON generation rate sensitivity simulation, we used $k_{Gly, \mathrm{NH}_{4}^{+}}^{\mathrm{II}}= {10}^{\left( 1.05-0.03 \right) pH_{cloud}-7.45}$ (unit:M^-1^ s^-1^), $k_{MG, \mathrm{NH}_{4}^{+}}^{\mathrm{II}}={10}^{\left( 0.834-0.2 \right) pH_{cloud}-5.91}$ (unit: M^-1^ s^-1^), and $k_{Gly, \mathrm{NH}_{4}^{+}}^{\mathrm{IV}}= 1.43-0.25\times{10}^{-11}\cdot{10}^{pH}$.

Figures S8 to S11 shows the simulated surface ON_fp_ and ON_p_ concentrations from the sensitivity simulations. Simulated global mean ON_fp_ concentrations were sensitive to both the N:C mass ratios for PON_p_ sources and the imine SON_p_ production rates. In the upper-limit simulation, the higher N:C emission ratios and the faster imine SON_p_ production increased the simulated global mean surface ON_fp_ concentration from 0.19 μg N m^-3^ to 0.32 μg N m^-3^, with a 0.05 μg N m^-3^ increase contributed by the primary emission and a 0.07 μg N m^-3^ increase contributed by faster imine SON generation. The resulting simulated ON_fp_ overestimated observations at most near-source sites and sites downwind of anthropogenic and biomass burning emissions (Fig. S8 A, B). In the lower-limit sensitivity simulation, the simulated global mean surface ON_fp_ concentration decreased from 0.19 μg N m^-3^ in the standard simulation to 0.06 μg N m^-3^, with a 0.11 μg N m^-3^ decrease from lower primary emissions (mainly due to less PON_p_ emissions from biomass burning) and a 0.02 μg N m^-3^ decrease contributed by slower imine SON_p_ production (Fig. S9 A, B). The lower-limit simulation underestimated the observed ON_fp_ concentrations at almost all locations. In the PON_p_-enhanced simulation, the simulated global mean surface ON_fp_ concentration only increased by 0.03 μg N m^-3^ (from 0.19 μg N m^-3^ to 0.22 μg N m^-3^), due to a 0.05 μg N m^-3^ increase of PON_p_ and a 0.02 μg N m^-3^ decrease of SON_p_ (Fig. S10 A, B). In the SON_p_-enhanced simulation, the simulated global mean surface ON_fp_ concentration decreased by 0.04 μg N m^-3^ (from 0.19 μg N m^-3^ to 0.15 μg N m^-3^) due to a 0.11 μg N m^-3^ decrease of PON_p_ and 0.07 μg N m^-3^ increase of SON_p_ (Fig. S11 A, B).

Based on our sensitivity simulations, on the global scale the most variable sources were biomass burning PON_p_ and imine SON_p_, whose contributions to the simulated global mean surface ON_fp_ concentration varied from 7% to 64% and from 9% to 74%, respectively. We found that the PON_p_-enhanced simulation and the SON_p_-enhanced simulation both reproduced the observed near-source to far-remote contrast of ON_p_ and ON_fp_ concentrations (Figs. S10, S11), indicating that both of these sources were important for global ON_fp_ abundance. Observations reported in the literature were deficient in distinguishing these two scenarios, because there were relatively few measurements in areas strongly affected by biomass burning and no explicit measurements of imine SON_p_ (Figs 2, S5, S10, S11, S12). As a result, our standard simulation may underestimate the contributions of biomass burning PON_p_ and SON precursors to global ON_p_ abundance, potentially up to a factor of 9.

In contrast to ON_fp_, the concentration of ON_cp_ was relatively stable and contributed less than 25% of the simulated global mean surface ON_p_ concentration in all sensitivity experiments with varying primary emissions and SON production rates. The simulated ON_cp_ mainly affected the simulated ON_p_ concentrations at marine sites, and the impacts of varying N:C mass ratios of ON­_cp_ were only obvious at marine sites. The simulated range of the atmospheric burden of ON was 1.09 to 1.51 Tg N, of which 0.09 to 0.51 Tg N was ON_p_. The simulated atmospheric burden of ON_p_ was composed of 0.07 to 0.46 Tg N of ON_fp_ and 0.02 to 0.05 Tg N of ON_cp_. Our sensitivity simulation showed that varying the N:C emission ratios and secondary production rates within the literature-reported ranges have large impacts on the simulated global budget of ON_fp_ but little impact on the simulated global budget of ON_cp_.

Figures S13 to S16 shows the simulated global atmospheric ON deposition fluxes from the sensitivity simulations. In the upper-limit simulation (Fig. S13), the global atmospheric ON deposition was 40 Tg N yr^-1^, with 2.5 Tg N yr^-1^ contributed by ON_g_, 28 Tg N yr^-1^ contributed by ON_fp_, and 9 Tg N yr^-1^ contributed by ON_cp_. In the lower-limit simulation (Fig. S14), the global atmospheric ON deposition was 10 Tg N yr^-1^, with 2.5 Tg N yr^-1^ contributed by ON_g_, 3.2 Tg N yr^-1^ contributed by ON_fp_, and 4.5 Tg N yr^-1^ contribute by ON_cp_. In the PON-enhanced simulation, the global atmospheric ON deposition was 28 Tg N yr^-1^, with only 1.8 Tg N yr^-1^ contributed by SON_p_ (Fig. S15). In the SON-enhanced simulation, the global ON deposition was 23 Tg N yr^-1^, with 14 Tg N yr^-1^ contributed by SON_p_ (Fig. S16).

For ON_fp_ deposition, the largest uncertainty was also associated with the formation of imine SON_p_, of which the global deposition flux ranged from 1.8 to 14 Tg N yr^-1^ and constituted 11% to 84% of the total ON_fp_ deposition in different combination. This was because imine SON_p_ was highly soluble, such that variation in the atmospheric abundance of imine SON_p_ would directly affect its deposition fluxes. The uncertainty associated with the N:C emission ratio from biomass burning also led to substantial variability in the simulated deposition flux (0.76 to 9.9 Tg N yr^-1^). For ON_cp_, although the choice of N:C emission ratios had little impacts on the simulated global mean ON_p_ concentration and burden, it significantly affected the global atmospheric ON_p_ deposition flux. The uncertainty of the global atmospheric deposition of ON_cp_ was mainly associated with the deposition of marine ON_cp_ (2 to 5 Tg N yr^-1^), followed by PBAPs (0.7 to 2 Tg N yr^-1^) and dust (0.6 to 2 Tg N yr^-1^). Gaseous ON was a minor contributor (2.5 Tg N yr^-1^) to the global ON deposition. We also find that, in the near-source regions with relatively higher deposition flux, the deposited ON_p_ were mainly from anthropogenic emissions, biomass burning emissions, and SON_p_ (Fig S17). Imine SON_p_ was the dominant contributor to our simulated SON_p_ deposition (Fig S17). ON_cp_ mainly contributed to ON deposition in remote areas, where the overall deposition flux of ON was relatively low. (Fig S17). Our upper-limit sensitivity simulation still underestimated the observed ON deposition fluxes by approximately a factor of 2, especially in high ON deposition areas affected by anthropogenic emission, biomass burning, and SON_p_ (Figs. S13, S17).

We conducted an additional sensitivity experiment by individually increasing the PON_p_ emissions from anthropogenic sources (Fig. S18A,D), from biomass burning (Fig. S18B,E), and the SON_p_ production rates (Fig. S18C, F), such that the simulated ON deposition flux fit the observation (i.e., reduced major axis regression line overlapped with the 1:1 line in Fig. S18). Compared to the standard simulation, we were able to simulate ON deposition fluxes consistent with the observations by increasing the anthropogenic emissions by 9 times, or by increasing biomass burning emissions by 5 times, or by increasing imine SON_p_ production by 5 times (Fig. S18). The correlations (*R* = 0.68) between the simulated and observed ON deposition fluxes was highest when biomass burning emissions were increased (Fig. S18). However, these experiments would all lead to systematic overestimation of the simulated ON_fp_ concentrations, especially the experiment where anthropogenic emissions were increased by a factor of 9. (Fig. S18). Based on all our sensitivity simulation, we concluded that our standard simulation may still underestimate the ON_p_ from biomass burning emissions and secondary production, but more measurements in regions affected by biomass burning are needed to better quantify the contributions from those sources.

**Text S3:** **Calculation of the contribution of ON to atmospherically deposited total bioavailable N to primary producers**

We calculated the total bioavailable N flux deposited from the atmosphere as the sum of bioavailable IN and bioavailable ON deposition from our standard ON simulation. IN is typically considered 100% bioavailable to primary producers, while the bioavailability of ON species differs. The percentage of ON mass that can be assimilated by primary producers ranged between 2% and 80% for bulk ON measurements from different sources [45-50]. Laboratory studies showed that ON species with small molecular size, low C:N ratios, and higher solubility tended to be more bioavailable [45, 48, 50]. In addition, phytoplankton communities appear to prefer reduced N because the reduced form N requires the least amount of energy to assimilate [51]. Due to the lack of explicit measurements of the bioavailability of ON from different primary and secondary sources, we used the measured ambient ON bioavailability at characteristic sites to represent the bioavailability of ON dominant at those sites. For example, we used the bioavailability of ON sampled at marine sites to represent the bioavailability of marine PON_p_ (Table S4). Imidazole in the particulate phase has been shown to be 100% bioavailable [52], and we thus assumed imine SON_p_ to be 100% bioavailable. There have not been direct measurements of the bioavailability of organic nitrate SON_p_ and NACs SON_p_; we assumed that they were 100% bioavailable on account of their hydrophilic qualities. The bioavailability of ON_g_ has also not been reported; we assumed the bioavailability of ON_g_ to be the average value (50%) of previously measured bulk ON. Overall, the insufficient constraints on the bioavailability of ON led to substantial uncertainty when assessing its ecosystem effects. Thus, we also conducted sensitivity calculations by assuming high-end and low-end values of ON bioavailability (Table S4) and applied them to the global atmospheric ON and TN deposition fluxes from our standard simulation.

Figure 6 shows the atmospheric deposition fluxes of bioavailable ON, the effective bioavailability of ON in the deposited ON fluxes, and the ratio of bioavailable ON versus bioavailable TN in the deposition fluxes. In the standard calculation (Fig. 6A-C), the global atmospheric deposition of bioavailable ON was 12 Tg N yr^-1^, which constituted 1% to 67% of the total bioavailable N deposition from the atmosphere. The effective bioavailability of ON in the deposited ON fluxes ranged from 25% to 91%. In the high-end ON bioavailability calculation (Fig. 6D-F), the global atmospheric deposition of bioavailable ON was 17 N yr^-1^, which constituted 2% to 76% of the total bioavailable N deposition from the atmosphere. The effective bioavailability of ON in deposited ON fluxes ranged from 36% to 96%. In the low-end ON bioavailability calculation (Fig. 6G-I), the global atmospheric deposition of bioavailable ON was 7 N yr^-1^, which constituted 0.8% to 51% of the total bioavailable N deposition from the atmosphere. The effective bioavailability of ON in the deposited ON fluxes ranged from 11% to 79%. We found that the spatial patterns of the ON deposition fluxes and the effective bioavailability of ON in deposited fluxes were similar in all sensitivity calculations, reflecting the regional disparity of ON and IN sources. As such, our qualitative assessments on the ecosystems where atmospheric ON deposition might be important was robust.


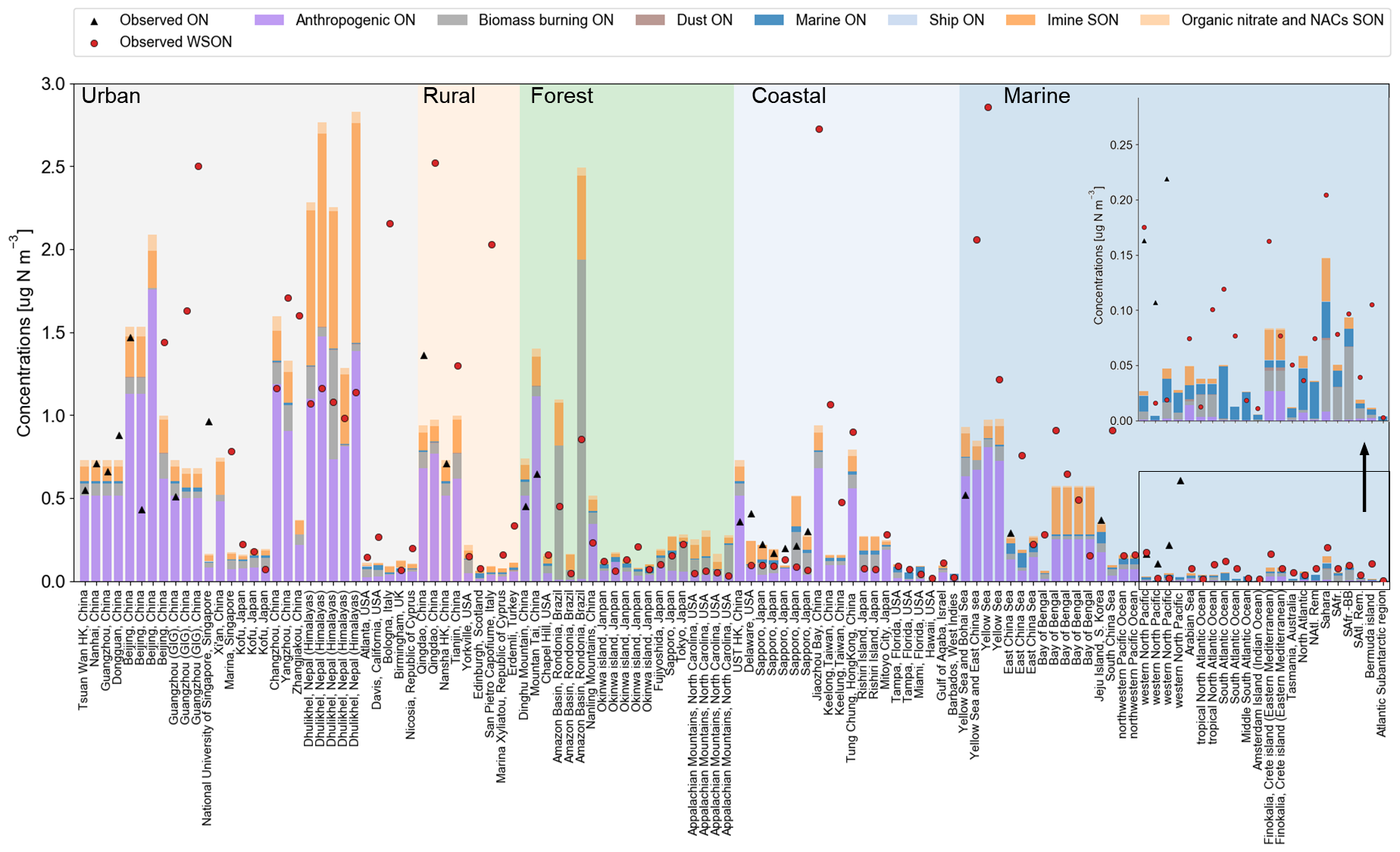


**Fig. S1. Site-by-site comparison of observed (ON: black triangles; WSON: red circles) and simulated (bars) ON_fp_ concentrations.** Simulated contributions from different ON_fp_ sources and the characteristics of observation sites are color-coded. References for the observations are summarized in Dataset S1.


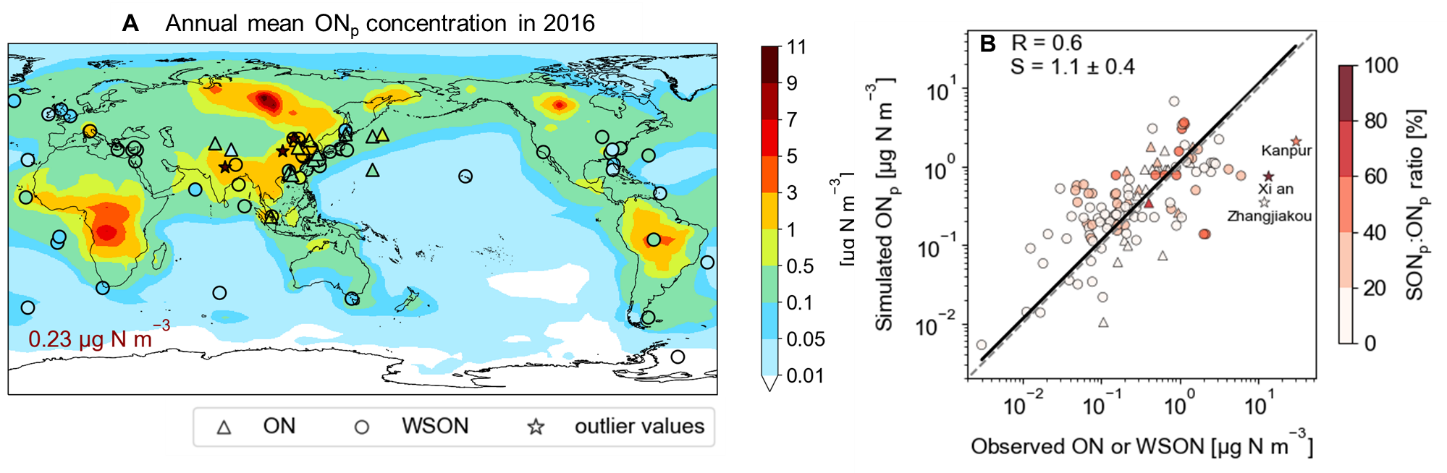


Fig. S2. Observed and simulated global annual mean surface ON_p_ concentrations. Observations shown as symbols: triangles: ON measurements; circles: water-soluble ON (WSON) measurements; stars: outliers affected by strong local, intermittent sources. (A) Observed and simulated (filled contours) annual mean surface ON_p_ concentrations. The simulated global annual mean surface ON_p_ concentration is shown inset. (B) Scatterplot of simulated versus observed annual mean surface ON_p_ concentrations, color-coded by the simulated SON_p_:ON_p_ ratios. Black line indicates the reduced major axis regression line, excluding the three outliers. Grey dashed line indicates the 1:1 line. The slope (S) and correlation coefficient (R) are shown inset.


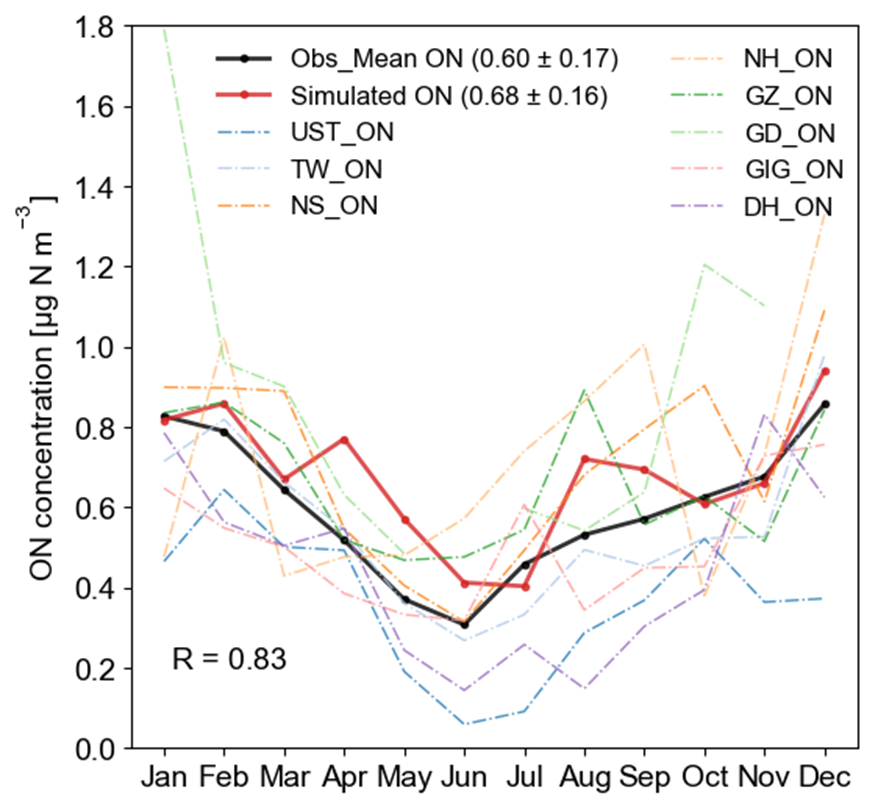


Fig. S3. Seasonal variation of observed (black and colored dash-dot lines) and simulated (red) ON_fp_ concentrations at eight sites in the Pearl River Delta area of southern China. The mean values and standard deviations of monthly observed and simulated ON_fp_ concentrations, as well as the correlation coefficient (R) between observed and simulated seasonal ON_fp_ concentrations, are shown inset.


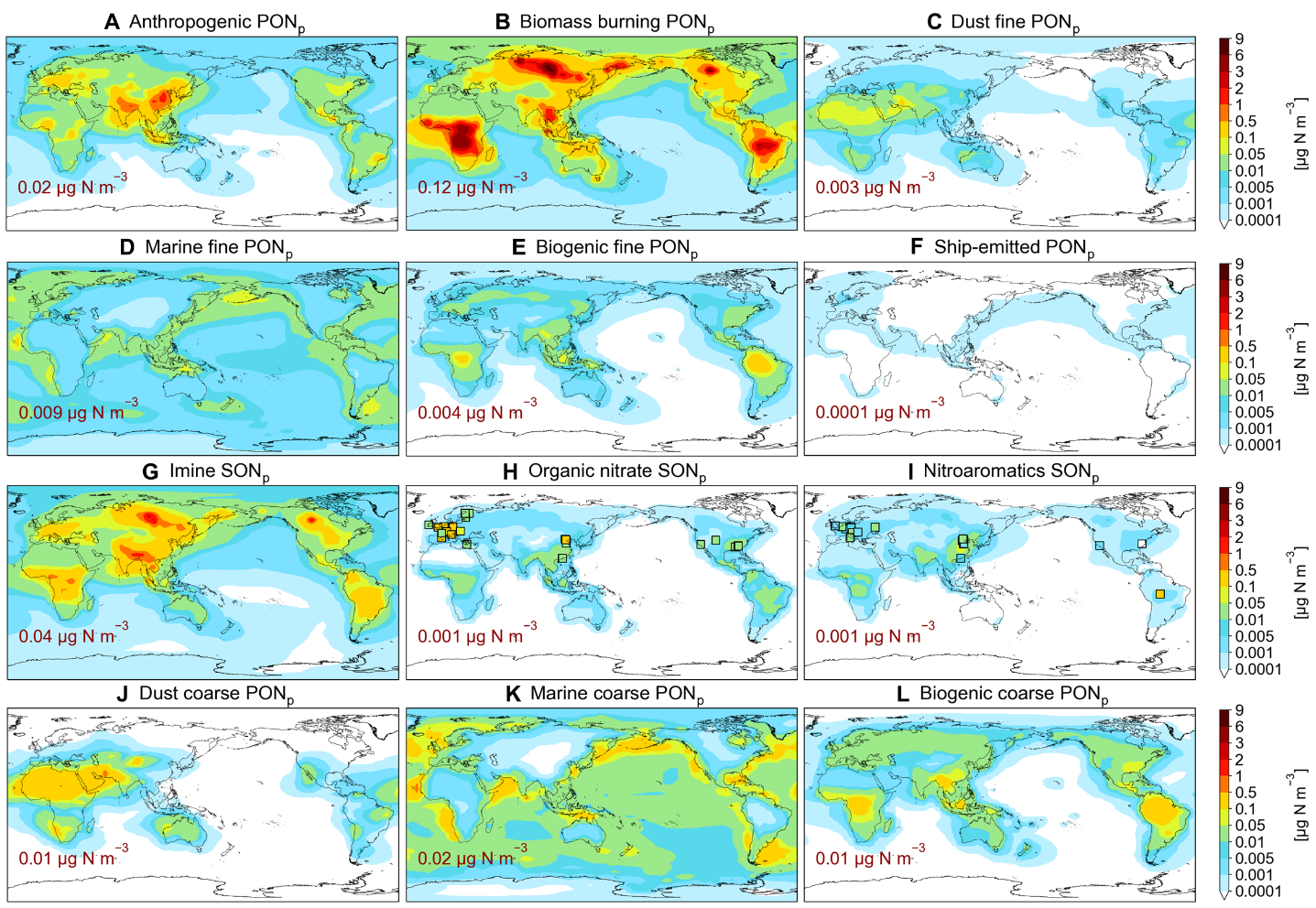


Fig. S4. Simulated global annual mean surface concentrations of ON_p_ from different sources. (A) anthropogenic PON_p_; (B) biomass burning PON_p_; (C) fine dust PON_p_; (D) fine marine PON_p_; (E) fine biogenic PON_p_; (F) ship-emitted PON_p_; (G) imine SON_p_; (H) organic nitrate SON_p_ and measurements of particulate organic nitrates; (I) nitroaromatic SON_p_ and measurements of particulate nitroaromatics; (J) coarse dust PON_p_; (K) coarse marine coarse PON_p_; (L) coarse biogenic PON_p_.


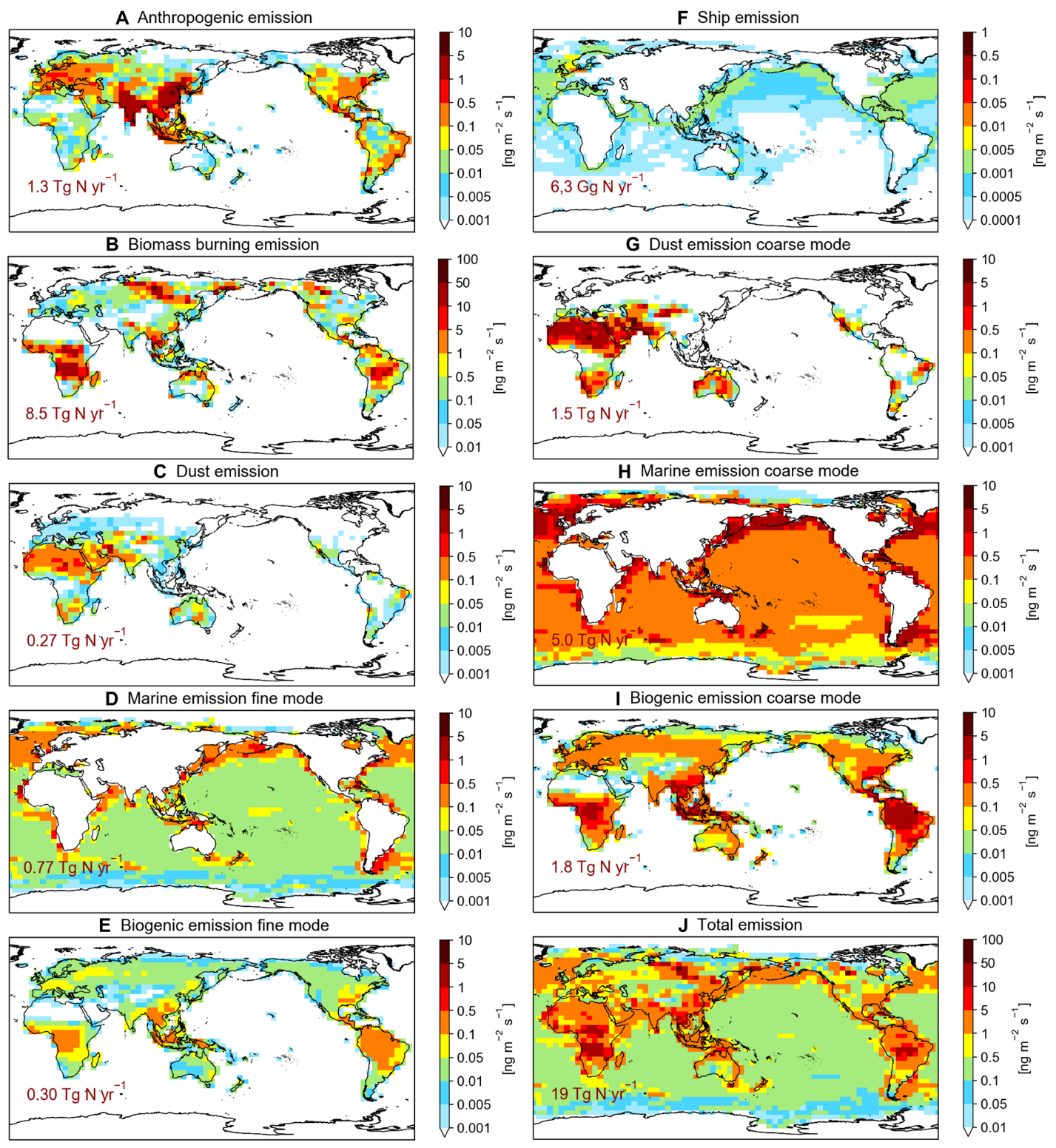


Fig. S5. Global emissions of PON_p_ from different sources for the year 2016. (A) anthropogenic PON_p_; (B) biomass burning PON_p_; (C) fine dust PON_p_; (D) fine marine PON_p_; (E) PON_p_ in fine primary biological atmospheric particles; (F) ship-emitted PON_p_; (G) coarse dust PON_p_; (H) coarse marine PON_p_; (I) PON_p_ in coarse biological atmospheric particles; (J) total PON_p_ emissions. The global annual emission totals from each source are shown inset.


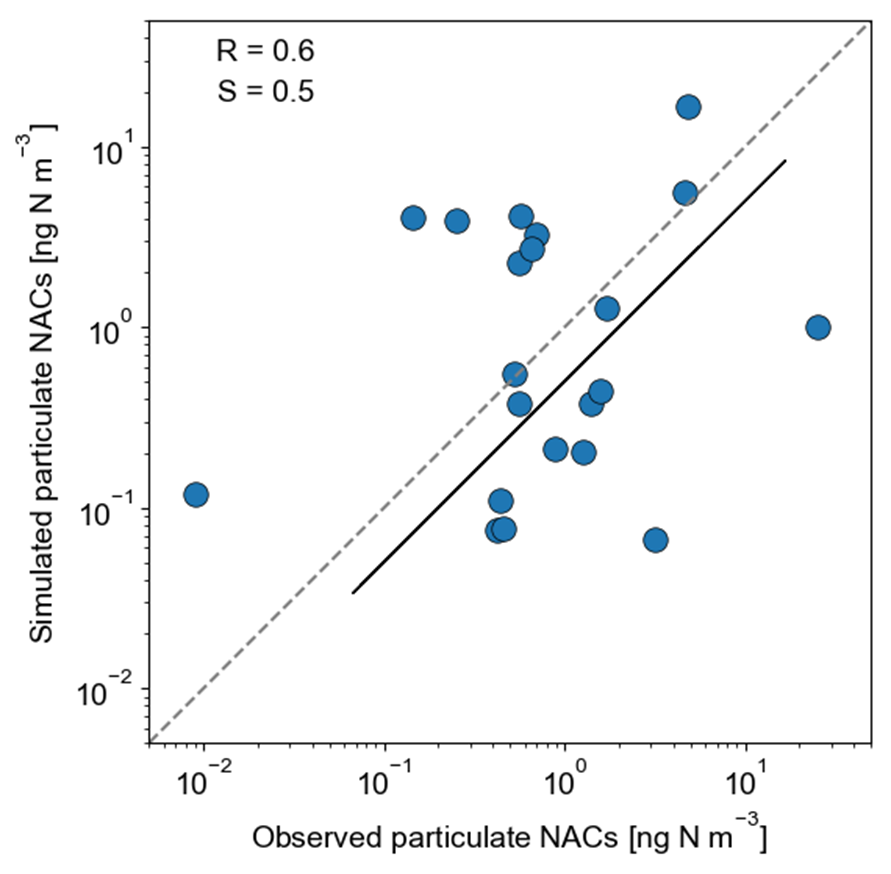


Fig. S6. Scatterplots of simulated and observed concentrations of particulate nitroaromatics (NACs). The black line indicates the reduced major axis regression line; the grey dashed line indicates the 1:1 line. References for the observations are given in Dataset S2.


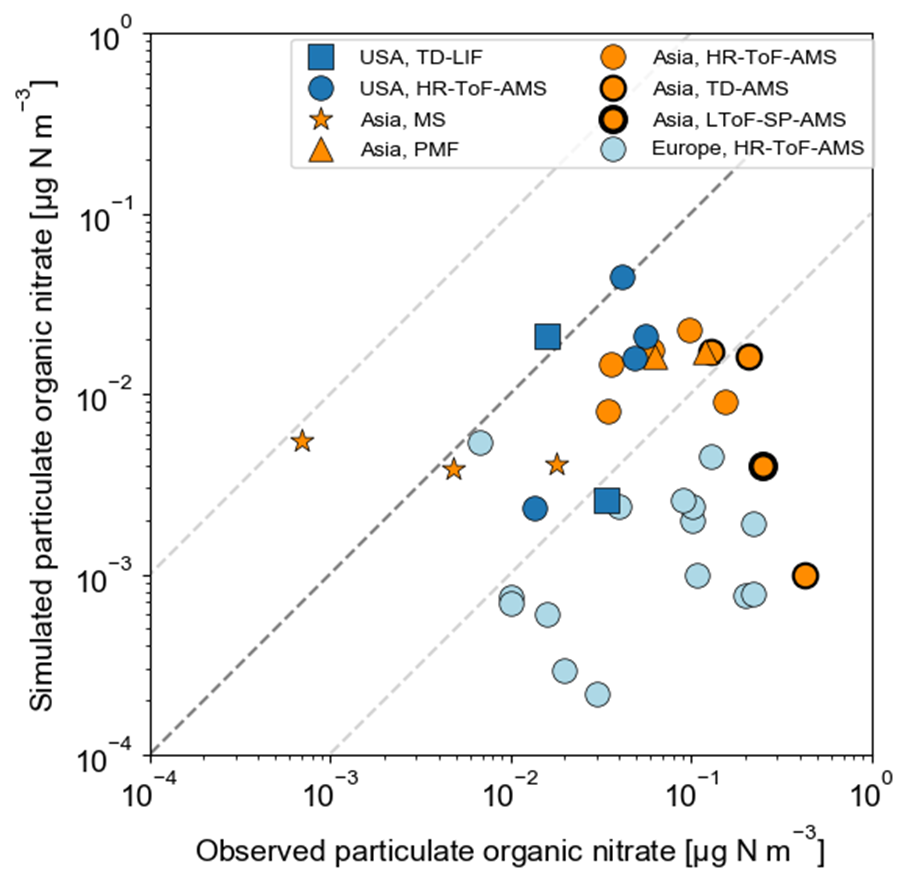


Fig. S7. Scatterplots of simulated and observed particulate organic nitrate concentrations. Blue symbols: observations in North America; orange symbols: observations in Asia; light blue symbols: observations in Europe. The dark grey dashed line indicates the 1:1 line; the light grey dashed lines indicate one order-of-magnitude differences. The observations are shape-coded by their measurement methods (details and references in Dataset S2).


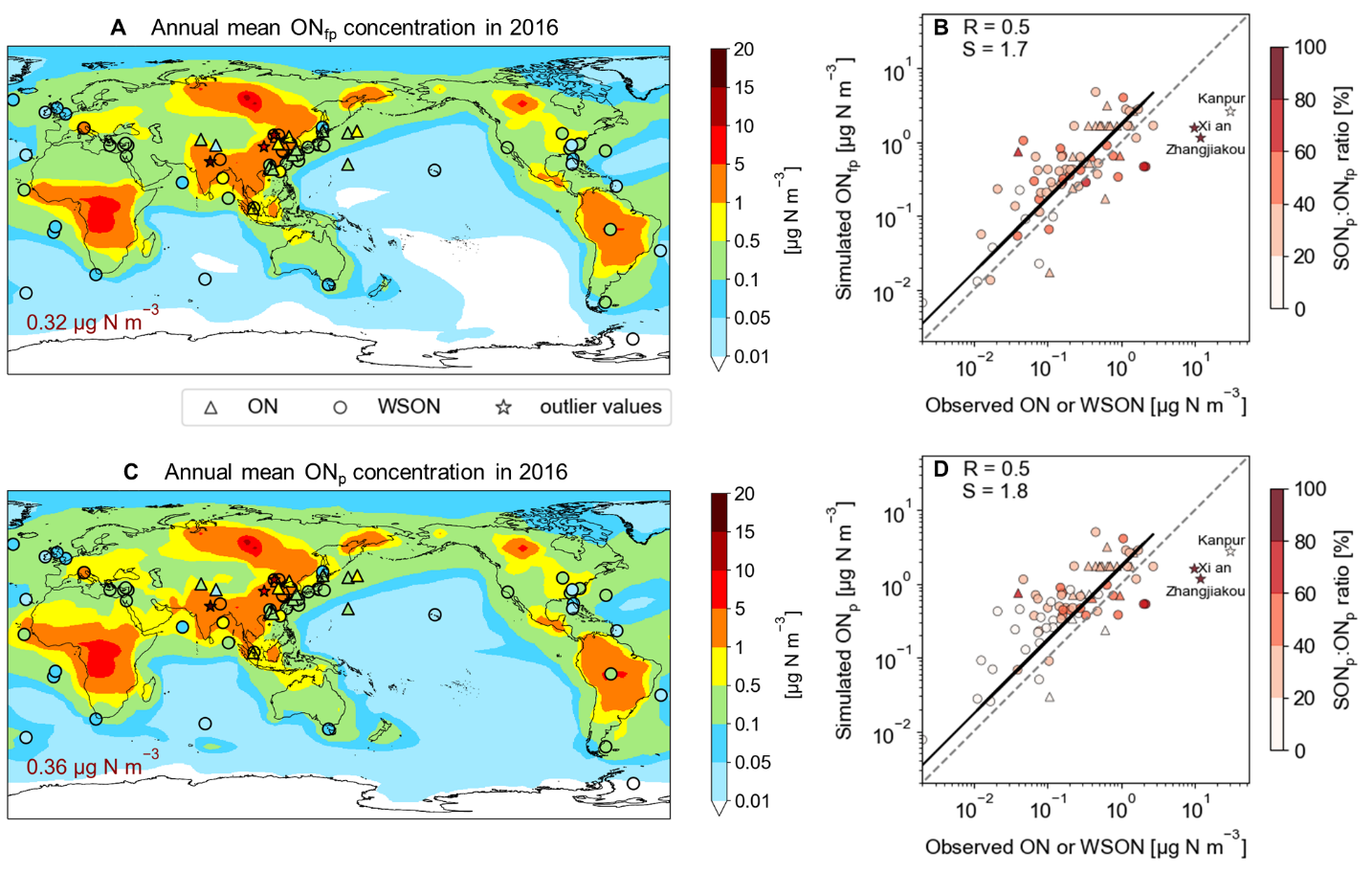


Fig. S8. Sensitivity simulation of global annual mean surface ON_fp_ and ON_p_ concentrations compared with observation, assuming upper-limit values of N:C emission ratios and fastest imine SON_p_ production. Observed data shown as symbols: triangles: ON measurements; circles: WSON measurements; stars: outliers affected by local, intermittent sources. (A, B) simulated ON_fp_ concentrations; (C, D) simulated ON_p_ concentrations. The simulated global annual mean surface ON_fp_ or ON_p_ concentrations are shown inset. The black lines indicate the reduced major axis regression lines; the grey dash lines indicate the 1:1 lines.


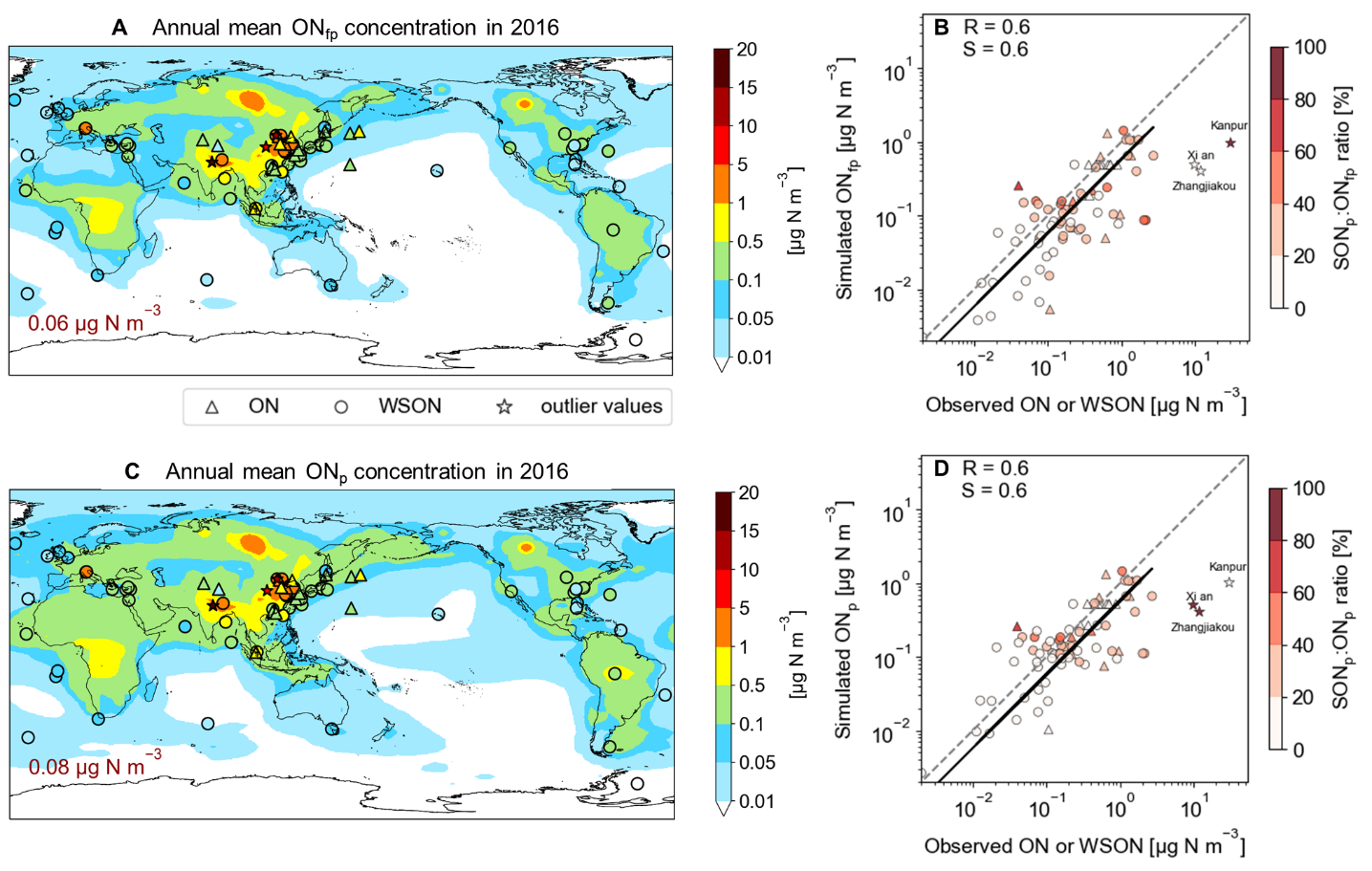


**Fig. S9. Sensitivity simulation of global annual mean surface ON_fp_ and ON_p_ concentrations compared with observation, assuming lower-limit values of N:C emission ratios and slowest imine SON_p_ production.** Observed data shown as symbols: triangles: ON measurements; circles: WSON measurements; stars: outliers affected by local, intermittent sources. **(A, B)** **s**imulated ON_fp_ concentrations; **(C, D)** simulated ON_p_ concentrations. The simulated global annual mean surface ON_fp_ or ON_p_ concentrations are shown inset. The black lines indicate the reduced major axis regression lines; the grey dash lines indicate the 1:1 lines.


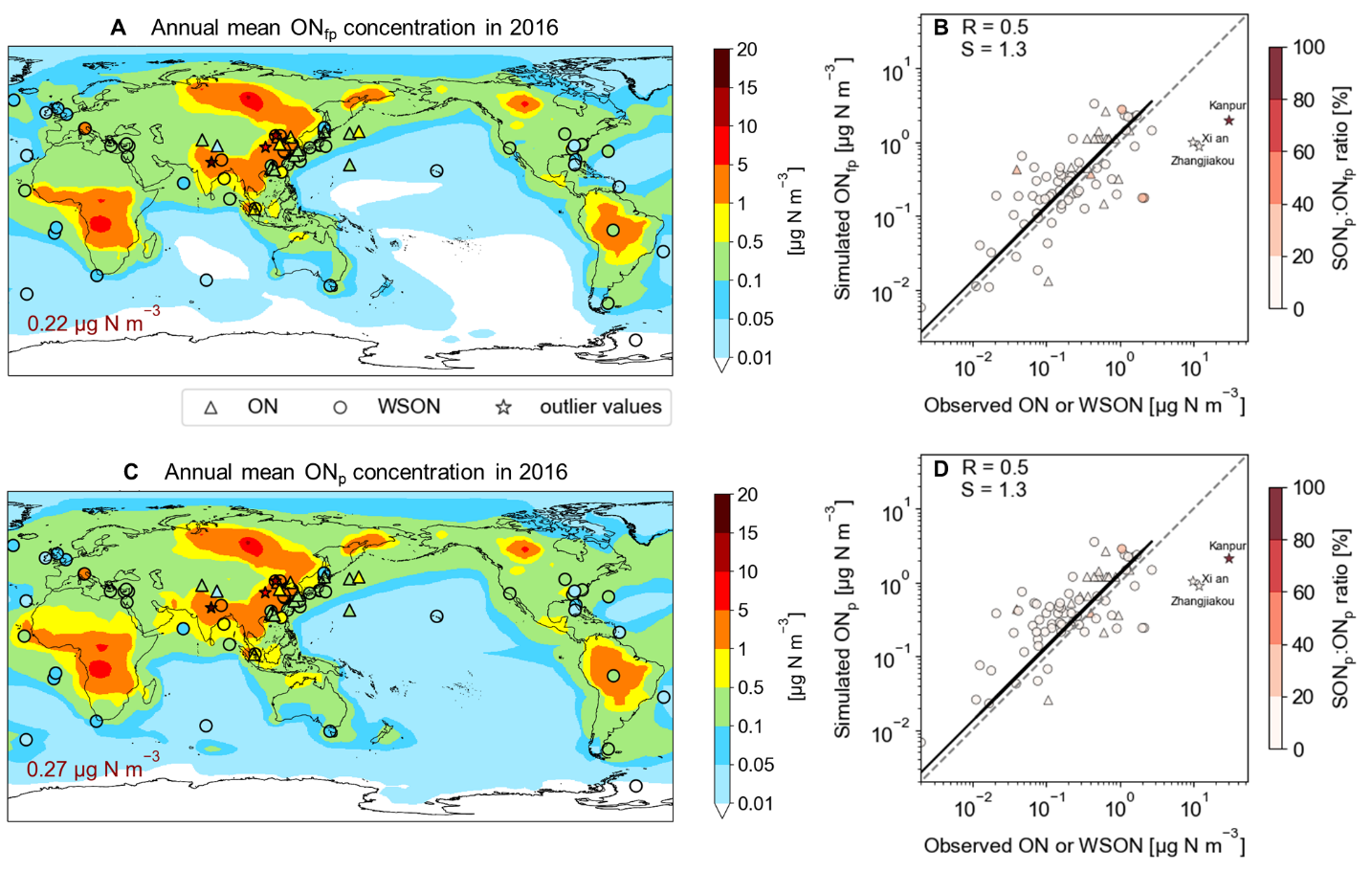


Fig. S10. Evaluation of a sensitivity simulation of global annual mean surface ON_fp_ and ON_p_ concentrations under a PON_p_-enhanced scenario, in which the simulation adopted upper-limit N:C emission ratios and lowest imine SON_p_ production rates. Observed data shown as symbols: triangles: ON measurements; circles: WSON measurements; stars: outliers affected by local, intermittent sources. (A, B) simulated ON_fp_ concentrations; (C, D) simulated ON_p_ concentrations. The simulated global annual mean surface ON_fp_ or ON_p_ concentrations are shown inset. The black lines indicate the reduced major axis regression lines; the grey dash lines indicate the 1:1 lines.


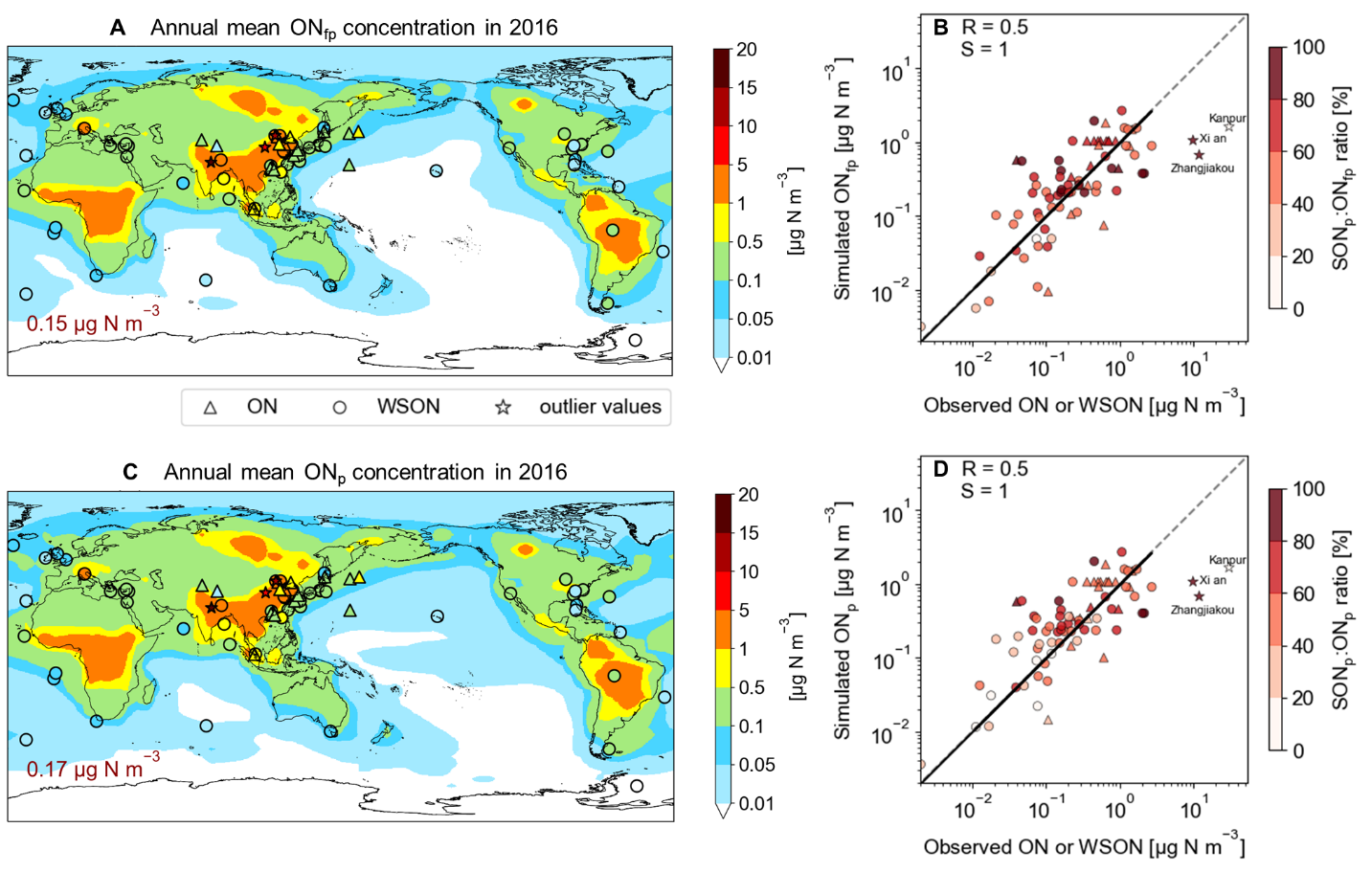


Fig. S11. Evaluation of a sensitivity simulation of global annual mean surface ON_fp_ and ON_p_ concentrations under an SON_p_-enhanced scenario, in which the simulation adopted lowest-limit N:C emission ratios and largest imine SON_p_ production rates. Observed data shown as symbols: triangles: ON measurements; circles: WSON measurements; stars: outliers affected by local, intermittent sources. (A, B) simulated ON_fp_ concentrations; (C, D) simulated ON_p_ concentrations. The simulated global annual mean surface ON_fp_ or ON_p_ concentrations are shown inset. The black lines indicate the reduced major axis regression lines; the grey dash lines indicate the 1:1 lines.


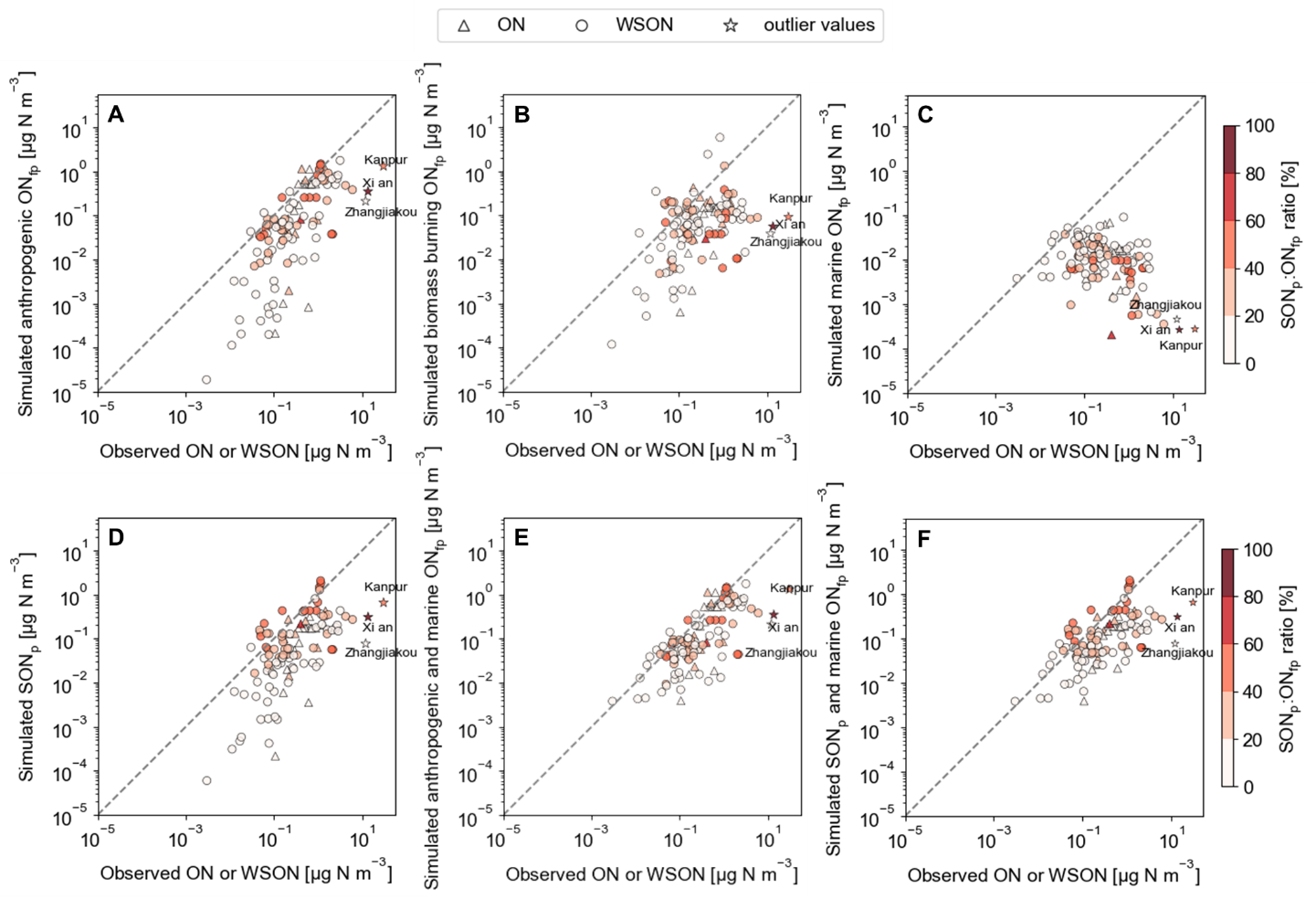


Fig. S12. Simulated concentrations of ON_fp_ from different primary and secondary sources from the standard simulation and their comparisons against the total observed ON concentrations. (A) simulated anthropogenic ON_fp_; (B) simulated biomass burning ON_fp_; (C) simulated marine ON_fp_; (D) simulated SON_p_; (E) simulated anthropogenic and marine ON_fp_; (F) simulated ON_fp_ and marine ON_fp_. Observed data shown as symbols: triangles: ON measurements; circles: WSON measurements; stars: outliers affected by local, intermittent sources. The colors of the symbols indicate the simulated total SON_p_:ON_fp_ fraction at each site in the standard simulation. The grey dashed line indicates the 1:1 line.


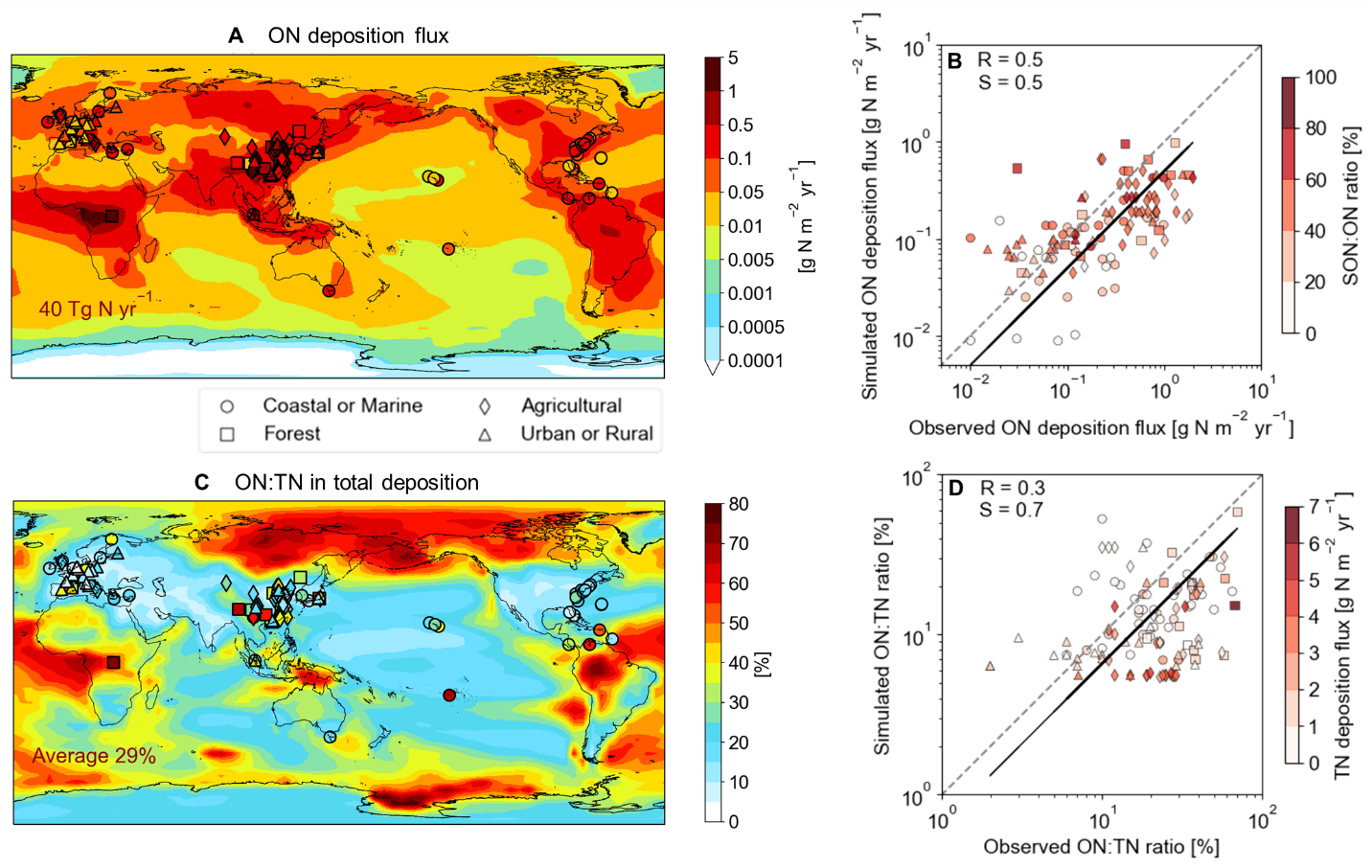


Fig. S13. Evaluation of the sensitivity simulation of ON deposition flux, assuming upper-limit N:C emission ratios and largest imine SON_p_ production. (A) Observed (symbols) and simulated (filled contours) ON deposition fluxes; (B) scatterplot of (A) and the reduced major axis regression (black line); (C) observed and simulated ON:TN ratios in atmospheric deposition fluxes; (D) scatterplot of (C). Observations shown in symbols: triangles: urban or rural sites; square: agricultural sites; diamond: forest sites; circles: marine or coastal sites. The global atmospheric ON deposition fluxes are shown inset. The black lines indicate the reduced major axis regression lines; the grey dash lines indicate the 1:1 lines.


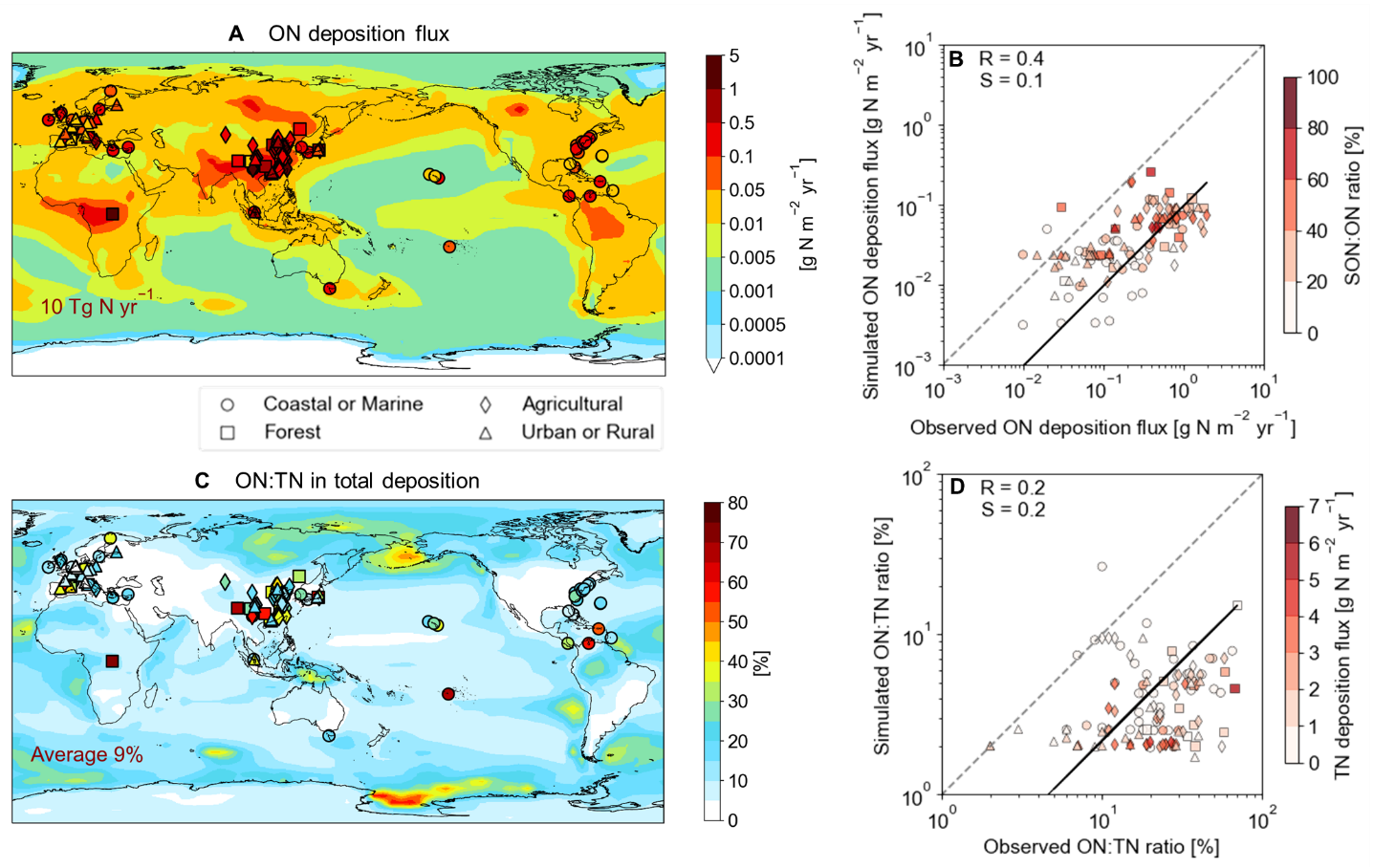


**Fig. S14. Evaluation of the sensitivity simulation of ON deposition flux, assuming lower-limit N:C emission ratios and slowest imine SON_p_ production.** (**A**) Observed (symbols) and simulated (filled contours) ON deposition fluxes; (**B**) scatterplot of (**A**) and the reduced major axis regression (black line); (**C**) observed and simulated ON:TN ratios in atmospheric deposition fluxes; (**D**) scatterplot of (**C**). Observations shown in symbols: triangles: urban or rural sites; square: agricultural sites; diamond: forest sites; circles: marine or coastal sites. The global atmospheric ON deposition fluxes are shown inset. The black lines indicate the reduced major axis regression lines; the grey dash lines indicate the 1:1 lines.


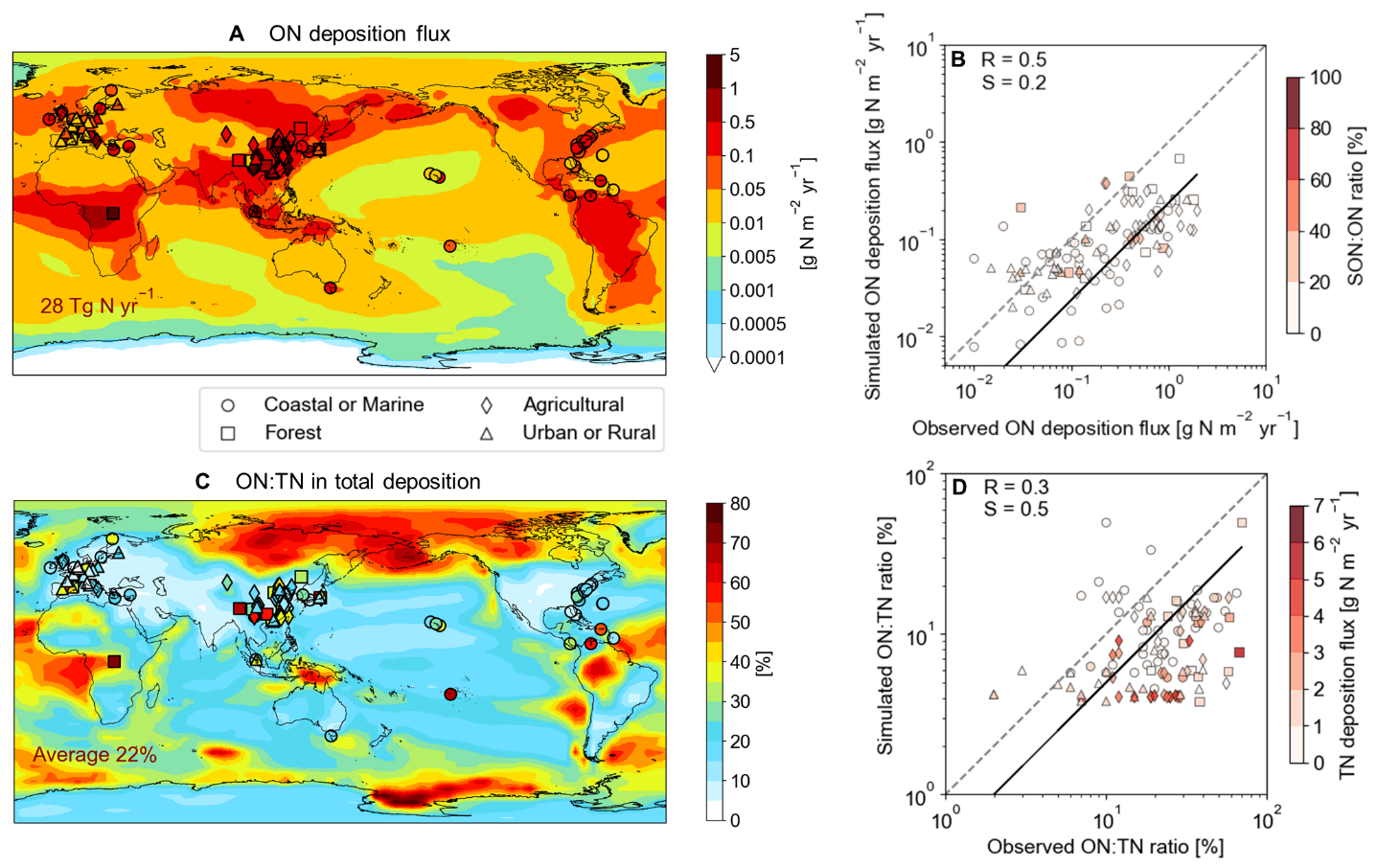


Fig. S15. Evaluation of the sensitivity simulation of ON deposition flux under a PON_p_-enhanced scenario, assuming upper-limit N:C emission ratios and lowest imine SON_p_ production. (A) observed (symbols) and simulated (filled contours) ON deposition fluxes; (B) scatterplot of (A); (C) observed and simulated ON:TN ratios in atmospheric deposition fluxes; (D) scatterplot of (C). Observations shown in symbols: triangles: urban or rural sites; square: agricultural sites; diamond: forest sites; circles: marine or coastal sites. The global atmospheric ON deposition fluxes are shown inset. The black lines indicate the reduced major axis regression lines; the grey dash lines indicate the 1:1 lines.


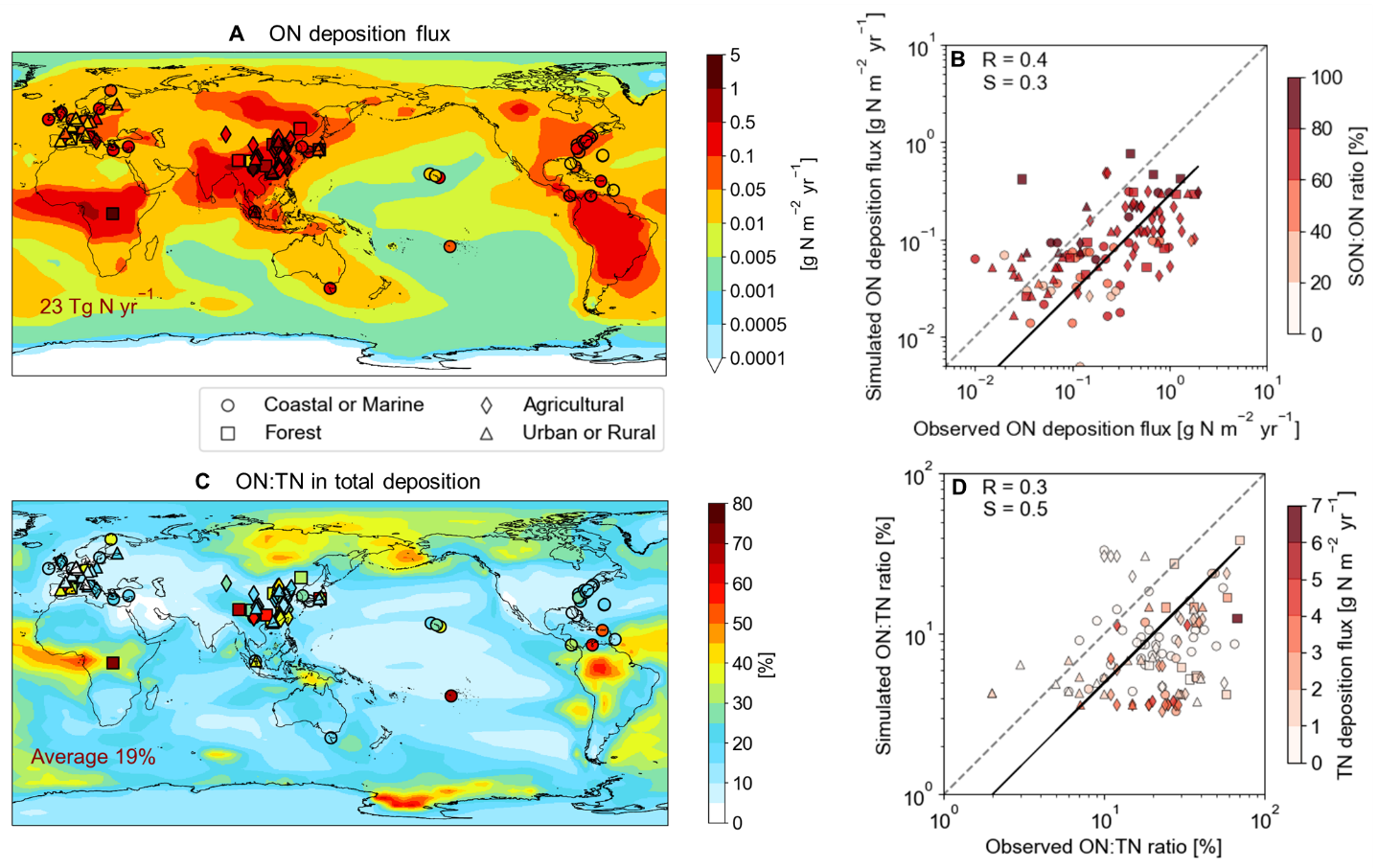


Fig. S16. Evaluation of the sensitivity simulation of ON deposition flux under an SON_p_-enhanced scenario, assuming lower-limit N:C emission ratios and largest imine SON_p_ production. (A) observed (symbols) and simulated (filled contours) ON deposition fluxes; (B) scatterplot of (A); (C) observed and simulated ON:TN ratios in atmospheric deposition fluxes; (D) scatterplot of (C). Observations shown in symbols: triangles: urban or rural sites; square: agricultural sites; diamond: forest sites; circles: marine or coastal sites. The global atmospheric ON deposition fluxes are shown inset. The black lines indicate the reduced major axis regression lines; the grey dash lines indicate the 1:1 lines.


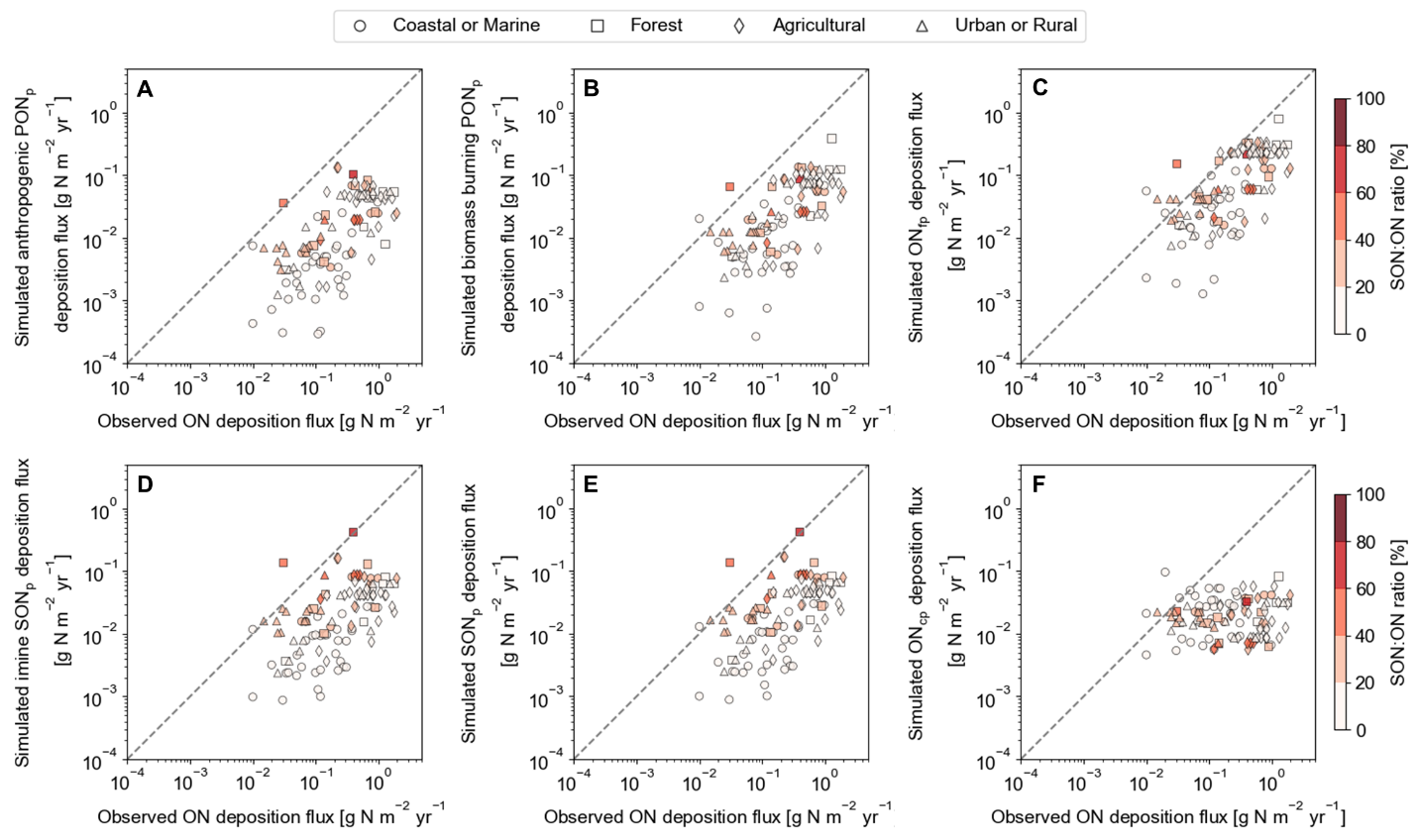


Fig. S17. Simulated deposition fluxes of ON_p_ from different sources in the standard simulation and their comparisons against the total observed ON deposition fluxes. (A) simulated anthropogenic PON_p_ deposition fluxes; (B) simulated biomass burning PON_p_ deposition fluxes; (C) simulated ON_fp_ deposition fluxes; (D) simulated imine SON_p_ deposition fluxes; (E) simulated SON_p_ deposition fluxes; (F) simulated ON_cp_ deposition fluxes. Observed data shown as symbols: triangles: ON measurements; circles: WSON measurements; stars: outliers affected by local, intermittent sources. The colors of the symbols indicate the simulated total SON:ON or SON_p_:ON_fp_ fractions at each site in the standard simulation. The grey lines indicate the 1:1 lines.


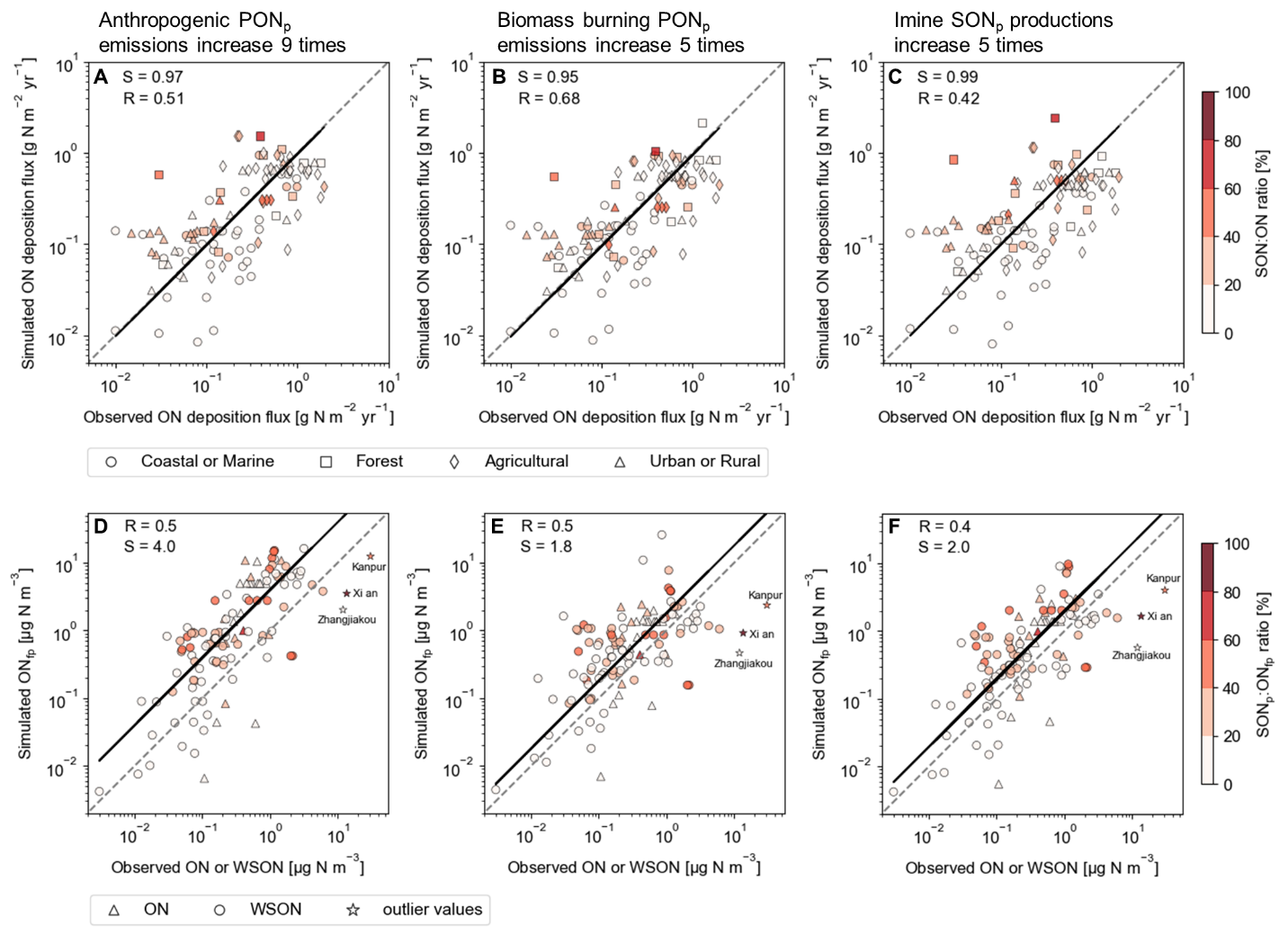


Fig. S18. Evaluation of sensitivity experiments in which (A, D) anthropogenic PON_p_ emissions, (B, E) biomass burning PON_p_ emissions, and (C, F) imine SON_p_ productions were individually increased to match the observed ON deposition fluxes. (A-C) simulated ON deposition fluxes compared with observations; (D-F) simulated ON_fp_ concentrations compared with observations. Observed data shown as symbols: triangles: ON measurements; circles: WSON measurements; stars: outliers affected by local, intermittent sources. The colors of the symbols indicate the simulated total SON:ON or SON_p_:ON_fp_ fractions at each site in the standard simulation. The grey lines indicate the 1:1 lines.

**Table S1. Summary of the source-specific N:C mass ratios and soluble fractions of the PON_p_ used in this study. The range of values used in the sensitivity simulations are shown in the parentheses.**

| **Sources** | **N:C mass ratio** | **PON_p_ soluble fraction** |
| --- | --- | --- |
| **Fine mode** |  |  |
| Anthropogenic PON_p_ | 0.08 (0.056-0.12)^a^ | 50%^c^ |
| Biomass burning PON_p_ | 0.60 (0.054-0.70)^b^ | 50%^c^ |
| Natural dust PON_p_ | 0.20 (0.081-0.26)^b^ | 10%^a,d^ |
| Anthropogenic fugitive combustion and industrial dust PON_p_ | 0.08 (0.056-0.12)^a^ | 50%^c^ |
| Primary biological particle PON_p_ | 0.15 (0.11-0.32)^b^ | 100%^e^ |
| Ship-emitted PON_p_ | 0.05 (0.04-0.09)^a^ | 50%^c^ |
| Marine PON_p_ | 0.15 (0.12-0.30)^b^ | 0%^f^ |
| **Coarse mode** |  |  |
| Marine PON_p_ | 0.30 (0.12-0.30)^b^ | 20%^b,f^ |
| Natural dust PON_p_ | 0.20 (0.081-0.26)^b^ | 10%^d^ |
| Primary biological particle PON_p_ | 0.30 (0.11-0.32)^b^ | 100%^e^ |

^a^ From our measurements based on ON_fp_ measurement [43].

^b^ From reference [4].

^c^ Set as the same as POC.

^d^ From reference [53].

^e^ Set as the same as OM from PBAPs.

^f^ Set as the same as OM from sea sprays.

Table S2. Summary of vapor pressures of semi-volatile nitroaromatic components.

| **Species** | **Vapor pressure under standard conditions (atm)** |
| --- | --- |
| Nitrophenol | 2.97×10^-7^ (6.6×10^-7^-1.5×10^-4^)^a,b^ |
| Nitrocatechol | 3.36×10^-10^ ^b^ |
| Methyl-nitrophenol | 4.15×10^-8^ (2.2×10^-9^-6.6×10^-8^)^a,b^ |

a From reference [21].

b From reference [17].

Table S3. Equilibrium constants for glyoxal and methylglyoxal with salt-in and salt-out effects, and RH effects in wet aerosols.

|  | **Glyoxal** | **Methylglyoxal** |
| --- | --- | --- |
| **Salting constants^a^** |  |  |
| $K_{{s,(NH_{4})}_{2}SO_{4}}$ | -0.24 | 0.16 |
| $K_{s},_{NH_{4}NO_{3}}$ | -0.07 | -0.075 |
|  |  |  |
| **RH acceleration coefficient^b^** |  |  |
| 35-45% | 4.0 | 1.3 |
| 45-55% | 4.4 | 1.7 |
| 55-65% | 4.0 | 1.7 |
| 65-75% | 2.4 | 1.4 |
| 75-84% | 1.2 | 1.1 |
| 84-100% | 1.0 | 1.0 |

^a^ Summarized by reference [30] from estimations.

^b^ Estimated by reference [34].

Table S4. Bioavailability of ON species from different sources

| **Sources** | **Bioavailability, *B*_ON_ [%]** |
| --- | --- |
|  |  |
| **ON** |  |
| **Primary ON_p_** |  |
| Anthropogenic | 59 ± 11^a^ |
| Biomass burning | 27 ± 17^b^ |
| Dust | 20 ± 10^c^ |
| Primary biological particle | 23 ± 19^d^ |
| Marine | 52 ± 23^e^ |
|  |  |
| **Secondary ON_p_** |  |
| Imine SON_p_ | 100^f^ |
| Organic nitrate SON_p_ | 100^f^ |
| NACs SON_p_ | 100^f^ |
|  |  |
| **ON_g_** | 50 ± 50^g^ |
|  |  |
| **IN** | 100^h^ |

^a^ From reference [45] measured in urban and suburban areas.

^b^ From reference [45] measured in forest and agricultural areas.

^c^ Assumed a low bioavailability based on the low hydrophilicity of ON from dust.

^d^ From reference [45] measured in forest areas.

^e^ From reference [47] and [48] measured in coastal and marine sites.

^f^ Assumed 100% bioavailability based on the high hydrophilicity of SON_p_ species and the observation that imidazole was highly bioavailable [46, 52].

^g^ Adopted the mean value from all ON bioavailability measurements.

^h^ Based on previous studies where IN is typically considered to be 100% bioavailable to marine microbes [54].

Dataset S1. (separate file)

Published measurements of atmospheric particulate organic nitrogen (ON_p_) and particulate water-soluble organic nitrogen (WSON_p_) concentrations at global surface sites.

Dataset S2. (separate file)

Published measurements of atmospheric particulate organic nitrates and particulate nitroaromatics concentrations at surface sites.

Dataset S3. (separate file)

Published measurements of atmospheric deposited organic nitrogen (ON) fluxes and the ratios of ON versus total nitrogen (ON:TN) in atmospheric deposition samples at global surface sites.

References

1. Bey I, Jacob DJ, Yantosca RM *et al.* Global modeling of tropospheric chemistry with assimilated meteorology: model description and evaluation. *Journal of Geophysical Research-Atmospheres*. 2001; **106**(D19): 23073-23095. doi: 10.1029/2001jd000807

2. Fountoukis C, Nenes A. ISORROPIA II: a computationally efficient thermodynamic equilibrium model for K+-Ca2+-Mg2+-Nh(4)(+)-Na+-SO42--NO3--Cl--H2O aerosols. *Atmospheric Chemistry and Physics*. 2007; **7**(17): 4639-4659. doi: 10.5194/acp-7-4639-2007

3. Heald CL, Spracklen DV. Atmospheric budget of primary biological aerosol particles from fungal spores. *Geophysical Research Letters*. 2009; **36**: 5. doi: Artn L0980610.1029/2009gl037493

4. Kanakidou M, Duce RA, Prospero JM *et al.* Atmospheric fluxes of organic N and P to the global ocean. *Global Biogeochemical Cycles*. 2012; **26**: 1-12. doi: 10.1029/2011gb004277

5. Ito A, Lin GX, Penner JE. Reconciling modeled and observed atmospheric deposition of soluble organic nitrogen at coastal locations. *Global Biogeochemical Cycles*. 2014; **28**(6): 617-630. doi: 10.1002/2013gb004721

6. Ito A, Lin GX, Penner JE. Global modeling study of soluble organic nitrogen from open biomass burning. *Atmospheric Environment*. 2015; **121**: 103-112. doi: 10.1016/j.atmosenv.2015.01.031

7. Yu X, Li Q, Liao K *et al.* New measurements reveal a large contribution of nitrogenous molecules to ambient organic aerosol. *ChemRxiv Cambridge: Cambridge Open Engage*. 2023. doi: 10.26434/chemrxiv-2023-ctldb

8. Ge XL, Wexler AS, Clegg SL. Atmospheric amines - part I. a review. *Atmospheric Environment*. 2011; **45**(3): 524-546. doi: 10.1016/j.atmosenv.2010.10.012

9. Perring AE, Pusede SE, Cohen RC. An observational perspective on the atmospheric impacts of alkyl and multifunctional nitrates on ozone and secondary organic aerosol. *Chemical Reviews*. 2013; **113**(8): 5848-5870. doi: 10.1021/cr300520x

10. Fisher JA, Jacob DJ, Travis KR *et al.* Organic nitrate chemistry and its implications for nitrogen budgets in an isoprene- and monoterpene-rich atmosphere: constraints from aircraft (SEAC^4^RS) and ground-based (SOAS) observations in the Southeast US. *Atmospheric Chemistry and Physics*. 2016; **16**(9): 5969-5991. doi: 10.5194/acp-16-5969-2016

11. Fisher JA, Atlas EL, Barletta B *et al.* Methyl, ethyl, and propyl nitrates: global distribution and impacts on reactive nitrogen in remote marine environments. *Journal of Geophysical Research-Atmospheres*. 2018; **123**(21): 12429-12451. doi: 10.1029/2018jd029046

12. Kiendler-Scharr A, Mensah AA, Friese E *et al.* Ubiquity of organic nitrates from nighttime chemistry in the European submicron aerosol. *Geophysical Research Letters*. 2016; **43**(14): 7735-7744. doi: 10.1002/2016gl069239

13. Ng NL, Brown SS, Archibald AT *et al.* Nitrate radicals and biogenic volatile organic compounds: oxidation, mechanisms, and organic aerosol. *Atmospheric Chemistry and Physics*. 2017; **17**(3): 2103-2162. doi: 10.5194/acp-17-2103-2017

14. Rollins AW, Pusede S, Wooldridge P *et al.* Gas/particle partitioning of total alkyl nitrates observed with TD-LIF in Bakersfield. *Journal of Geophysical Research-Atmospheres*. 2013; **118**(12): 6651-6662. doi: 10.1002/jgrd.50522

15. Bates KH, Jacob DJ. A new model mechanism for atmospheric oxidation of isoprene: global effects on oxidants, nitrogen oxides, organic products, and secondary organic aerosol. *Atmospheric Chemistry and Physics*. 2019; **19**(14): 9613-9640. doi: 10.5194/acp-19-9613-2019

16. Harrison MAJ, Barra S, Borghesi D *et al.* Nitrated phenols in the atmosphere: a review. *Atmospheric Environment*. 2005; **39**(2): 231-248. doi: 10.1016/j.atmosenv.2004.09.044

17. Wang Y, Hu M, Wang Y *et al.* The formation of nitro-aromatic compounds under high NO_x_ and anthropogenic VOC conditions in urban Beijing, China. *Atmospheric Chemistry and Physics*. 2019; **19**(11): 7649-7665. doi: 10.5194/acp-19-7649-2019

18. Andreae MO. Emission of trace gases and aerosols from biomass burning - an updated assessment. *Atmospheric Chemistry and Physics*. 2019; **19**(13): 8523-8546. doi: 10.5194/acp-19-8523-2019

19. Ng NL, Kroll JH, Chan AWH *et al.* Secondary organic aerosol formation from m-xylene, toluene, and benzene. *Atmospheric Chemistry and Physics*. 2007; **7**(14): 3909-3922. doi: 10.5194/acp-7-3909-2007

20. Bloss C, Wagner V, Jenkin ME *et al.* Development of a detailed chemical mechanism (MCMv3.1) for the atmospheric oxidation of aromatic hydrocarbons. *Atmospheric Chemistry and Physics*. 2005; **5**: 641-664. doi: 10.5194/acp-5-641-2005

21. Yuan B, Liggio J, Wentzell J *et al.* Secondary formation of nitrated phenols: insights from observations during the Uintah Basin Winter Ozone Study (UBWOS) 2014. *Atmospheric Chemistry and Physics*. 2016; **16**(4): 2139-2153. doi: 10.5194/acp-16-2139-2016

22. Cecinato A, Di Palo V, Pomata D *et al.* Measurement of phase-distributed nitrophenols in Rome ambient air. *Chemosphere*. 2005; **59**(5): 679-683. doi: 10.1016/j.chemosphere.2004.10.045

23. Fu T-M, Jacob DJ, Wittrock F *et al.* Global budgets of atmospheric glyoxal and methylglyoxal, and implications for formation of secondary organic aerosols. *Journal of Geophysical Research-Atmospheres*. 2008; **113**(D15). doi: 10.1029/2007jd009505

24. Noziere B, Dziedzic P, Cordova A. Products and kinetics of the liquid-phase reaction of glyoxal catalyzed by ammonium ions (NH_4_^+^). *Journal of Physical Chemistry A*. 2009; **113**(1): 231-237. doi: 10.1021/jp8078293

25. De Haan DO, Hawkins LN, Kononenko JA *et al.* Formation of nitrogen-containing oligomers by methylglyoxal and amines in simulated evaporating cloud droplets. *Environmental Science & Technology*. 2011; **45**(3): 984-991. doi: 10.1021/es102933x

26. Yu G, Bayer AR, Galloway MM *et al.* Glyoxal in aqueous ammonium sulfate solutions: products, kinetics and hydration effects. *Environmental Science & Technology*. 2011; **45**(15): 6336-6342. doi: 10.1021/es200989n

27. Kua J, Krizner HE, De Haan DO. Thermodynamics and kinetics of imidazole formation from glyoxal, methylamine, and formaldehyde: A computational study. *Journal of Physical Chemistry A*. 2011; **115**(9): 1667-1675. doi: 10.1021/jp111527x

28. Sedehi N, Takano H, Blasic VA *et al.* Temperature- and pH-dependent aqueous-phase kinetics of the reactions of glyoxal and methylglyoxal with atmospheric amines and ammonium sulfate. *Atmospheric Environment*. 2013; **77**: 656-663. doi: 10.1016/j.atmosenv.2013.05.070

29. Galloway MM, Powelson MH, Sedehi N *et al.* Secondary organic aerosol formation during evaporation of droplets containing atmospheric aldehydes, amines, and ammonium sulfate. *Environmental Science & Technology*. 2014; **48**(24): 14417-14425. doi: 10.1021/es5044479

30. Curry LA, Tsui WG, McNeill VF. Technical note: updated parameterization of the reactive uptake of glyoxal and methylglyoxal by atmospheric aerosols and cloud droplets. *Atmospheric Chemistry and Physics*. 2018; **18**(13): 9823-9830. doi: 10.5194/acp-18-9823-2018

31. Shah V, Jacob DJ, Moch JM *et al.* Global modeling of cloud water acidity, precipitation acidity, and acid inputs to ecosystems. *Atmospheric Chemistry and Physics*. 2020; **20**(20): 12223-12245. doi: 10.5194/acp-20-12223-2020

32. Alexander B, Allman DJ, Amos HM *et al.* Isotopic constraints on the formation pathways of sulfate aerosol in the marine boundary layer of the subtropical northeast Atlantic Ocean. *Journal of Geophysical Research-Atmospheres*. 2012; **117**. doi: 10.1029/2011jd016773

33. Rodriguez AA, de Loera A, Powelson MH *et al.* Formaldehyde and acetaldehyde increase aqueous-phase production of imidazoles in methylglyoxal/amine mixtures: quantifying a secondary organic aerosol formation mechanism. *Environmental Science & Technology Letters*. 2017; **4**(6): 234-239. doi: 10.1021/acs.estlett.7b00129

34. Kasthuriarachchi NY, Rivellini L-H, Chen X *et al.* Effect of relative humidity on secondary brown carbon formation in aqueous droplets. *Environmental Science & Technology*. 2020; **54**(20): 13207-13216. doi: 10.1021/acs.est.0c01239

35. Fischer EV, Jacob DJ, Yantosca RM *et al.* Atmospheric peroxyacetyl nitrate (PAN): a global budget and source attribution. *Atmospheric Chemistry and Physics*. 2014; **14**(5): 2679-2698. doi: 10.5194/acp-14-2679-2014

36. Browne EC, Perring AE, Wooldridge PJ *et al.* Global and regional effects of the photochemistry of CH3O2NO2: evidence from ARCTAS. *Atmospheric Chemistry and Physics*. 2011; **11**(9): 4209-4219. doi: 10.5194/acp-11-4209-2011

37. Wang Y, Hu M, Li X *et al.* Chemical composition, sources and formation mechanisms of particulate brown carbon in the atmosphere. *Progress in Chemistry*. 2020; **32**(5): 627-641. doi: 10.7536/pc190917

38. Zhao R, Lee AKY, Huang L *et al.* Photochemical processing of aqueous atmospheric brown carbon. *Atmospheric Chemistry and Physics*. 2015; **15**(11): 6087-6100. doi: 10.5194/acp-15-6087-2015

39. Sumlin BJ, Pandey A, Walker MJ *et al.* Atmospheric photooxidation diminishes light absorption by primary brown carbon aerosol from biomass burning. *Environmental Science & Technology Letters*. 2017; **4**(12): 540-545. doi: 10.1021/acs.estlett.7b00393

40. Lee HJ, Aiona PK, Laskin A *et al.* Effect of solar radiation on the optical properties and molecular composition of laboratory proxies of atmospheric brown carbon. *Environmental Science & Technology*. 2014; **48**(17): 10217-10226. doi: 10.1021/es502515r

41. Liu J, Lin P, Laskin A *et al.* Optical properties and aging of light-absorbing secondary organic aerosol. *Atmospheric Chemistry and Physics*. 2016; **16**(19): 12815-12827. doi: 10.5194/acp-16-12815-2016

42. Singh GK, Rajeev P, Paul D *et al.* Chemical characterization and stable nitrogen isotope composition of nitrogenous component of ambient aerosols from Kanpur in the Indo-Gangetic Plains. *Science of the Total Environment*. 2021; **763**. doi: 10.1016/j.scitotenv.2020.143032

43. Yu X, Li Q, Ge Y *et al.* Simultaneous determination of aerosol inorganic and organic nitrogen by thermal evolution and chemiluminescence detection. *Environmental Science & Technology*. 2021; **55**(17): 11579-11589. doi: 10.1021/acs.est.1c04876

44. Jickells T, Baker AR, Cape JN *et al.* The cycling of organic nitrogen through the atmosphere. *Philosophical Transactions of the Royal Society B-Biological Sciences*. 2013; **368**(1621). doi: 10.1098/rstb.2013.0115

45. Seitzinger SP, Sanders RW, Styles R. Bioavailability of DON from natural and anthropogenic sources to estuarine plankton. *Limnology and Oceanography*. 2002; **47**(2): 353-366. doi: 10.4319/lo.2002.47.2.0353

46. Petrone KC, Richards JS, Grierson PF. Bioavailability and composition of dissolved organic carbon and nitrogen in a near coastal catchment of south-western Australia. *Biogeochemistry*. 2009; **92**(1-2): 27-40. doi: 10.1007/s10533-008-9238-z

47. Xiu B, Liang S-k, He X-l *et al.* Bioavailability of dissolved organic nitrogen and its uptake by Ulva prolifera: implications in the outbreak of a green bloom off the coast of Qingdao, China. *Marine Pollution Bulletin*. 2019; **140**: 563-572. doi: 10.1016/j.marpolbul.2019.01.057

48. Li M, Li KQ, Chen K *et al.* Size-based bioavailability of land-based DON and its impact on eutrophication of Jiaozhou bay. *Marine Pollution Bulletin*. 2020; **152**. doi: 10.1016/j.marpolbul.2020.110898

49. Zhu Q, Zhuang Q. Modeling the effects of organic nitrogen uptake by plants on the carbon cycling of boreal forest and tundra ecosystems. *Biogeosciences*. 2013; **10**(12): 7943-7955. doi: 10.5194/bg-10-7943-2013

50. Zhai TE, Zhang JT, Huo SL *et al.* Algal activity of dissolved organic nitrogen (DON) in the sediments of Lake Taihu, Eastern China. *Environmental Earth Sciences*. 2016; **75**(24). doi: 10.1007/s12665-016-6286-x

51. Bronk DA, See JH, Bradley P *et al.* DON as a source of bioavailable nitrogen for phytoplankton. *Biogeosciences*. 2007; **4**(3): 283-296. doi: 10.5194/bg-4-283-2007

52. Verma A, Joshi S, Singh D. Imidazole: having versatile biological activities. *Journal of Chemistry*. 2013; **2013**. doi: 10.1155/2013/329412

53. Park SS, Cho SY. Characterization of organic aerosol particles observed during Asian dust events in spring 2010. *Aerosol and Air Quality Research*. 2013; **13**(3): 1019-1033. doi: 10.4209/aaqr.2012.06.0142

54. Soares ARA, Bergstrom AK, Sponseller RA *et al.* New insights on resource stoichiometry: assessing availability of carbon, nitrogen, and phosphorus to bacterioplankton. *Biogeosciences*. 2017; **14**(6): 1527-1539. doi: 10.5194/bg-14-1527-2017
